# SARS-CoV-2-specific CD8^+^ T cells from people with long COVID establish and maintain effector phenotype and key TCR signatures over 2 years

**DOI:** 10.1073/pnas.2411428121

**Published:** 2024-09-16

**Authors:** Louise C. Rowntree, Jennifer Audsley, Lilith F. Allen, Hayley A. McQuilten, Ruth R. Hagen, Priyanka Chaurasia, Jan Petersen, Dene R. Littler, Hyon-Xhi Tan, Lydia Murdiyarso, Jennifer R. Habel, Isabelle J. H. Foo, Wuji Zhang, Elizabeth R. V. ten Berge, Hanujah Ganesh, Prathanporn Kaewpreedee, Kelly W. K. Lee, Samuel M. S. Cheng, Janette S. Y. Kwok, Dhilshan Jayasinghe, Stephanie Gras, Jennifer A. Juno, Adam K. Wheatley, Stephen J. Kent, Jamie Rossjohn, Allen C. Cheng, Tom C. Kotsimbos, Jason A. Trubiano, Natasha E. Holmes, Ken Ka Pang Chan, David S. C. Hui, Malik Peiris, Leo L. M. Poon, Sharon R. Lewin, Peter C. Doherty, Irani Thevarajan, Sophie A. Valkenburg, Katherine Kedzierska, Thi H. O. Nguyen

**Affiliations:** ^a^Department of Microbiology and Immunology, University of Melbourne, at the Peter Doherty Institute for Infection and Immunity, Melbourne, VIC 3000, Australia; ^b^Department of Infectious Diseases, University of Melbourne, at the Peter Doherty Institute for Infection and Immunity, Melbourne, VIC 3000, Australia; ^c^Infection and Immunity Program and Department of Biochemistry and Molecular Biology, Biomedicine Discovery Institute, Monash University, Clayton, VIC 3800, Australia; ^d^HKU-Pasteur Research Pole, School of Public Health, The University of Hong Kong, Hong Kong Special Administrative Region, China; ^e^Division of Public Health Laboratory Sciences, School of Public Health, The University of Hong Kong, Hong Kong Special Administrative Region, China; ^f^Division of Transplantation and Immunogenetics, Department of Pathology, Queen Mary Hospital, Hong Kong Special Administrative Region, China; ^g^Infection & Immunity Program, La Trobe Institute for Molecular Science, La Trobe University, Bundoora, VIC 3083, Australia; ^h^Department of Biochemistry and Chemistry, School of Agriculture, Biomedicine and Environment, La Trobe University, Bundoora, VIC 3083, Australia; ^i^Institute of Infection and Immunity, Cardiff University School of Medicine, Cardiff CF14 4XN, United Kingdom; ^j^School of Public Health and Preventive Medicine, Monash University, Melbourne, VIC 3004, Australia; ^k^Monash Infectious Diseases, Monash Health and School of Clinical Sciences, Monash University, Clayton, VIC 3168, Australia; ^l^Department of Respiratory Medicine, The Alfred Hospital, Melbourne, VIC 3004, Australia; ^m^Department of Medicine, Central Clinical School, The Alfred Hospital, Monash University, Melbourne, VIC 3004, Australia; ^n^Department of Infectious Diseases, Peter MacCallum Cancer Centre, Melbourne, VIC 3000, Australia; ^o^National Centre for Infections in Cancer, Peter McCallum Cancer Centre, Melbourne, VIC 3000, Australia; ^p^Department of Medicine (Austin Health), University of Melbourne, Heidelberg, VIC 3084, Australia; ^q^Centre for Antibiotic Allergy and Research, Department of Infectious Diseases, Austin Health, Heidelberg, VIC 3084, Australia; ^r^Department of Critical Care, University of Melbourne, Parkville, VIC 3000, Australia; ^s^Data Analytics Research and Evaluation Centre, Austin Health and University of Melbourne, Heidelberg, VIC 3084, Australia; ^t^Department of Medicine and Therapeutics, Prince of Wales Hospital, The Chinese University of Hong Kong, Hong Kong Special Administrative Region, China; ^u^Li Ka Shing Institute of Health Sciences, Faculty of Medicine, The Chinese University of Hong Kong, Hong Kong Special Administrative Region, China; ^v^Centre for Immunology and Infection, Hong Kong Science and Technology Park, New Territories, Hong Kong Special Administrative Region, China; ^w^Victorian Infectious Diseases Service, Royal Melbourne Hospital, at the Peter Doherty Institute for Infection and Immunity, Melbourne, VIC 3000, Australia; ^x^Department of Infectious Disease, Alfred Hospital and Monash University, Melbourne, VIC 3000, Australia

**Keywords:** T cells, SARS-CoV-2 epitopes, long COVID, T cell receptors

## Abstract

Long COVID occurs in a small but important minority of patients following COVID-19, reducing quality of life and contributing to healthcare burden. Although research into underlying mechanisms is evolving, immunity is understudied. SARS-CoV-2-specific T cell responses are of key importance for viral clearance and COVID-19 recovery. However, in long COVID, the establishment and persistence of SARS-CoV-2-specific T cells are far from clear, especially beyond 12 mo postinfection and postvaccination. We defined ex vivo antigen-specific B cell and T cell responses and their T cell receptors (TCR) repertoires across 2 y postinfection in people with long COVID. Using 13 SARS-CoV-2 peptide–HLA tetramers, spanning 11 HLA allotypes, as well as spike and nucleocapsid probes, we tracked SARS-CoV-2-specific CD8^+^ and CD4^+^ T cells and B-cells in individuals from their first SARS-CoV-2 infection through primary vaccination over 24 mo. The frequencies of ORF1a- and nucleocapsid-specific T cells and B cells remained stable over 24 mo. Spike-specific CD8^+^ and CD4^+^ T cells and B cells were boosted by SARS-CoV-2 vaccination, indicating immunization, in fully recovered and people with long COVID, altered the immunodominance hierarchy of SARS-CoV-2 T cell epitopes. Meanwhile, influenza-specific CD8^+^ T cells were stable across 24 mo, suggesting no bystander-activation. Compared to total T cell populations, SARS-CoV-2-specific T cells were enriched for central memory phenotype, although the proportion of central memory T cells decreased following acute illness. Importantly, TCR repertoire composition was maintained throughout long COVID, including postvaccination, to 2 y postinfection. Overall, we defined ex vivo SARS-CoV-2-specific B cells and T cells to understand primary and recall responses, providing key insights into antigen-specific responses in people with long COVID.

## Significance

Long COVID occurs in small but important minority of patients following COVID-19, reducing quality of life and contributing to healthcare burden. Although research into underlying mechanisms is evolving, immunity is understudied. As the recall of T cell memory promotes more rapid recovery and ameliorates disease outcomes, establishment of robust memory T cells is important for protection against subsequent infections, even when the virus mutates. We defined how SARS-CoV-2-specific T cell and B cell responses are established and maintained following infection and vaccination for 2 y in people with long COVID. We found robust and prototypical SARS-CoV-2-specific T cells with effector phenotype and key T cell receptor signatures in people with long COVID following SARS-CoV-2 infection and subsequent COVID-19 vaccination.

Long COVID, or post-acute sequelae of COVID-19, is an ongoing global health burden. Long COVID impacts multiple organ systems including respiratory, cardiovascular, gastrointestinal, endocrine, and neurological systems and affected individuals report a wide array of symptoms that can persist anywhere from 12 wk up to 2 y or longer post COVID-19 diagnosis (1, 2). Most common symptoms are weakness and fatigue, impaired concentration, myalgia, and chest pain, with many more varied symptoms being reported (3–5). Prevalence of long COVID varies widely between cohorts, with studies describing anywhere from below 10% to over 60% of patients reporting continuing symptoms more than 12 wk after SARS-CoV-2 infection, with higher percentages found in hospitalized COVID-19 patients (6–9). It has been observed that the proportion of individuals reporting one or more long-term post-COVID symptoms decreases over time, from 68% at 6 mo to 55% at 24 mo (10). Although research into the underlying mechanisms is evolving and multiple causes have been proposed, there is no one explanation that demonstrates clear causality. Long COVID may instead be attributed to multiple nonmutually exclusive mechanisms that together account for the similarly large spectrum of clinical symptoms.

Proposed mechanisms to explain long COVID prevalence include ongoing presence of organ damage caused by SARS-CoV-2 infection and the associated immune response (11–13), reactivation of latent Epstein–Barr virus and associated mononucleosis (14, 15), microbiome dysfunction (16), persistence of a viral reservoir somewhere in the body despite clearance in the nasopharyngeal tract (17), purely a suboptimal (either subdued or excessive) immune response to the original infection (18) or inflammatory pathways related to tissue damage (19). Long COVID may develop due to a combination of mechanisms. Continuing long COVID symptoms at six months have been associated with a weak SARS-CoV-2 anti-spike (20) and anti-nucleocapsid (21) IgG antibody response at the peak of infection, as well as with acute and persistent complement activation in the serum (22).

SARS-CoV-2-specific CD4^+^ and CD8^+^ T cells play an important role in viral clearance and recovery from COVID-19 (23–25). However, recruitment and persistence of T cells, together with any potential perturbations, are less understood in long COVID. Previous studies examined total CD4^+^ and CD8^+^ T cell responses (26, 27) or T cells directed toward SARS-CoV-2 peptide pools (28, 29) in long COVID. People with long COVID recovering from severe COVID-19 showed exhausted and activated T cell populations up to 8 mo after infection (27, 29, 30). Wiech et al. found the CD8^+^ T cell population differentiated toward an effector memory CD45RA^+^ T cell (Temra) phenotype at 3 to 6 mo post infection in long COVID, characterizing a prolonged cytotoxic phenotype (27). People with long COVID had lower frequencies of total CD4^+^ and CD8^+^ effector memory T cells (Tem) circulating in the blood at 2 to 12 mo post-COVID-19 (26). Following stimulation with overlapping peptide pools, long COVID individuals showed prolonged activation of SARS-CoV-2-specific CD4^+^ and CD8^+^ T cells compared to fully recovered patients, with increased proportions of circulating T cells readily producing IFN-γ and TNF (28, 30, 31) at 4 mo. Similarly, SARS-CoV-2-specific CD8^+^ T cell responses from individuals with long COVID preferentially expressed exhaustion markers PD-1 and CTLA4 compared to fully recovered individuals at 8 mo (31). Exhaustion/activation markers PD-1 and TIM-3 on both CD4^+^ and CD8^+^ T cells did not persist at 24 mo (32).

Introduction of COVID-19 vaccines further complicated efforts to understand long COVID. Although COVID-19 vaccination reduced the risk of SARS-CoV-2 infection, long COVID rates are not significantly reduced in vaccinated individuals (33). Breakthrough infections following vaccination show similar prevalence of long COVID, with conflicting studies reporting either worsened or improved long COVID symptoms after vaccination (34–36). Thus, long COVID still constitutes a substantial health problem even in the era of COVID-19 vaccination, and understanding long-term persistence of SARS-CoV-2-specific T cells and B cells after infection and vaccination is of key importance.

Using an established long COVID cohort, we addressed a knowledge gap in understanding antigen-specific T cell and B cell responses over 2 y post first COVID-19 diagnosis, and COVID-19 vaccine impact on T and B cell responses in long COVID. Using 13 SARS-CoV-2 peptide–HLA tetramers, and spike and nucleocapsid B cell probes, we assessed antigen-specific T cells and B cells directly ex vivo. We defined how SARS-CoV-2-specific T cells and B cells are established and maintained after infection and vaccination for two years in individuals with long COVID, in comparison to fully recovered individuals. We found that SARS-CoV-2-specific CD4^+^ and CD8^+^ T cells from people with long COVID establish and maintain prominent SARS-CoV-2-specific T cell pools with effector phenotype and key TCR signatures over 2 y. Our study defines ex vivo SARS-CoV-2-specific T cells during primary and recall responses and provides key insights into CD8^+^ and CD4^+^ T cell responses in fully recovered and long COVID people, following SARS-CoV-2 infection and subsequent COVID-19 vaccination.

## Results

### Longitudinal Analysis of Long COVID and Non-LC Cohorts.

**Fig. 1.**
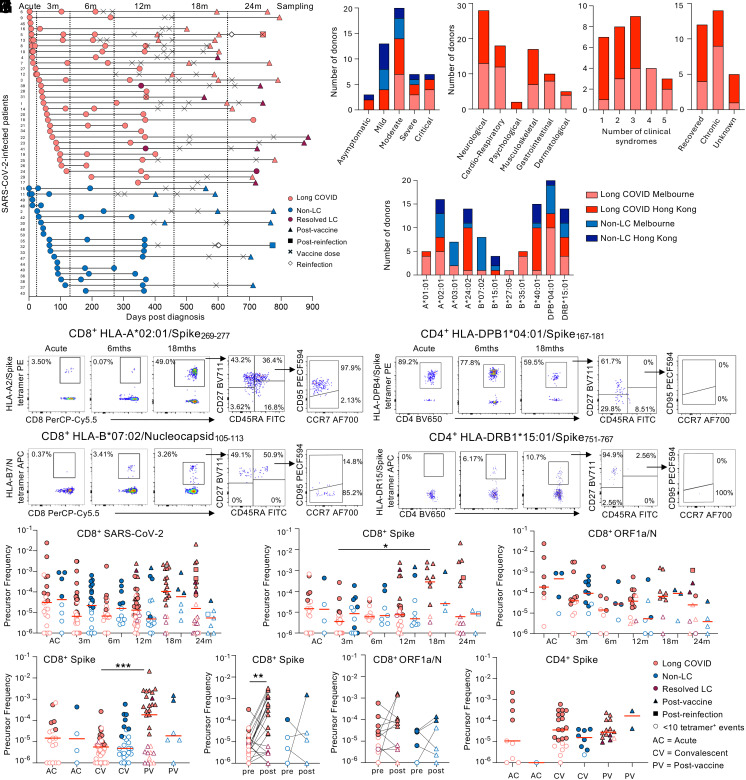
SARS-CoV-2-specific CD8^+^ and CD4^+^ T cell responses in people with long COVID. (*A*) Sampling timepoints. (*B*) Severity of initial SARS-CoV-2 infection. (*C*) Clinical syndromes, number of clinical syndromes, and study endpoint recovery status of people with long COVID. (*D*) HLA distribution across participants. (*E*) Donor representative FACS plots of TAME enriched SARS-CoV-2-specific CD8^+^ and CD4^+^ T cells. (*F*) CD8^+^ SARS-CoV-2-specific and (*G*) CD8^+^ spike-specific and CD8^+^ ORF1a/N-specific tetramer^+^ T cell frequencies in people with long COVID and non-LC controls over time post diagnosis. 7 datapoints are from previously described COVID-19 adult cohort (39). (*H*) CD8^+^ spike-specific T cell precursor frequencies grouped by acute, convalescent, and post-COVID-19 vaccination. (*I, i*) Paired CD8^+^ spike-specific and (*I, ii*) CD8^+^ ORF1a/N-specific T cell frequencies pre- and post-SARS-CoV-2-vaccination. (*J*) CD4^+^ spike-specific T cell precursor frequencies grouped by acute, convalescent, and post-COVID-19 vaccination. Statistical significance determined by (*F–H* and *J*) Dunn’s multiple comparisons test comparing all timepoints, (*I*) Wilcoxon matched-pairs sign-rank test for comparison between pre- and postvaccination. (*F–I*) Frequency of tetramer^+^ cells has been shifted up by 10^−6^ (i.e. samples with zero tetramer^+^ events displayed as 10^−6^) for visibility on the logarithmic y-axis. Samples with <10 tetramer^+^ events are shown as open symbols.

We longitudinally analyzed 31 people with long COVID who had been HLA-typed, for up to 24 mo post-acute SARS-CoV-2 infection (Fig. 1*A* and *SI Appendix*, Table S1). Individuals were designated as long COVID in accordance with the WHO definition of symptoms >3 mo post SARS-CoV-2 infection, last for at least 2 mo and cannot be explained by an alternative diagnosis (37). Participants were part of two prospective cohort, one recruited in Melbourne (Australia) in hospitals and the other in Hong Kong quarantine, both from February to October 2020. At the same time, nineteen individuals without ongoing symptoms were recruited and included as non-long COVID (non-LC) controls. Longitudinal samples were grouped across acute infection (days 0 to 23, median 9 d long COVID/7 d non-LC), ~3 mo (days 24 to 129, median 68/68 d), ~6 mo (days 130 to 269, median 209/180 d), ~12 mo (days 270 to 459, median 367/365 d), ~18 mo (days 460 to 629, median 593/591 d) and ~24 mo (days 630 to 889, median 743/767 d) (Fig. 1*A*). Our study included individuals across a range of acute COVID-19 severities, with 33% and 25% severe/critical cases, 47% and 33% moderate, and 20% and 42% asymptomatic/mild acute SARS-CoV-2 infections in long COVID and non-LC groups, respectively (Fig. 1*B*). Of individuals sampled 18 to 24 mo postinfection, all but two people with long COVID received at least one SARS-CoV-2 vaccine dose (average time of first vaccine postinfection was 432 d (range 226 to 635) for long COVID and 461 d (282 to 594) for non-LC) (Fig. 1*A*). One person with long COVID and one non-LC control reported known SARS-CoV-2 reinfection following vaccination prior to their final study visit (24 mo). The most common reported long COVID clinical syndrome was neurological (90%), including fatigue, headache, memory issues, lack of concentration (“mental fog”), mental confusion, dizziness, tremor, paresthesia, loss of taste or smell, problems speaking, and balance issues (Fig. 1*C*). Of individuals with long COVID, more than one clinical syndrome was reported by 77% (24/31) (Fig. 1*C*). Meanwhile 52% (16/31) were affected by ≥3 clinical syndromes, and of those, 38% recovered by their final study visit. Overall, 40% of people with long COVID self-reported being fully recovered by study endpoint (Fig. 1*C*). For epitope-specific T cell analyses, we determined frequencies of individuals expressing at least one HLA allele with known SARS-CoV-2 T cell epitopes within our personalized tetramer panel, with high prevalence of class I HLA-A*02:01 (32%), HLA-A*24:02 (28%), HLA-B*40:01 (30%), and class II DPB1*04:01 (40%), DRB1*15:01 (28%) observed (Fig. 1*D*).

### Prototypical SARS-CoV-2 Epitope-Specific CD8^+^ and CD4^+^ T Cell Responses in People with Long COVID Across 24 mo.

To define longitudinal circulating SARS-CoV-2 epitope-specific T cells in people with long COVID, we used SARS-CoV-2 peptide–HLA tetramers to assess T cell responses directly ex vivo against 11 CD8^+^ (A1/ORF1a_1637_, A1/S_865_, A2/S_269,_ A3/N_361_, A3/S_378,_ A24/S_1208_, B7/N_105_, B15/S_919_, B27/N_9_, B35/S_321_, and B40/N_322_) and two CD4^+^ (DPB4/S_167_ and DR15/S_751_) SARS-CoV-2 epitopes (Fig. 1*E* and *SI Appendix*, Fig. S1*A*) (38–43). These epitopes encompass peptides highly conserved across variants of concern (VOC) strains, while B15/S_919_ is cross-reactive with common cold coronaviruses (HKU1/OC43) (40, 41, 44).

Prototypical and robust populations of circulating SARS-CoV-2 epitope-specific CD8^+^ and CD4^+^ T cells were observed at all timepoints, starting from acute disease up to 24 mo post SARS-CoV-2 infection in people with long COVID and non-LC controls (Fig. 1*F* and *SI Appendix*, Fig. S2 *A* and *B*). The mean frequency of SARS-CoV-2 epitope-specific CD8^+^ T cells was stable across time. When data were stratified according to spike (A1/S_865_, A2/S_269,_ A3/S_378,_ A24/S_1208_, B15/S_919_, and B35/S_321_) and nonspike (A1/ORF1a_1637_, A3/N_361_, B7/N_105_, B27/N_9_, and B40/N_322_) CD8^+^ T cell specificities, frequency of spike-specific CD8^+^ T cells in people with long COVID increased from 1.98 × 10^−5^ at 3-mo to 7.47 × 10^−4^ at 18 mo post SARS-CoV-2 infection (3.77 fold increase, *P* = 0.0126), while ORF1a- and N-specific CD8^+^ T cell responses remained unchanged (Fig. 1*G*). The increase in spike-specific CD8^+^ T cells at 18 mo was maintained when repeated measures per epitope (multiple samples per donor within a time grouping) were averaged (*P* = 0.036) (*SI Appendix*, Fig. S2*C*). Meanwhile, spike-specific CD4^+^ T cell responses remained stable over time in people with and without long COVID (*SI Appendix*, Fig. S2*A*).

To ensure the increase in spike-specific CD8^+^ T cells was the result of vaccination and not function of time postinfection, frequency of epitope-specific CD8^+^ T cells was compared between acute infection, during vaccine-naïve convalescence (3 to 24 mo postinfection) and postvaccination convalescence (12 to 24 mo). Spike-specific CD8^+^ T cell frequencies in people with long COVID showed a dip at vaccine-naïve convalescence, followed by a significant increase postvaccination (*P* = 0.0012) (Fig. 1*H*), which trended toward significance when repeated measures were averaged (*SI Appendix*, Fig. S2*D*). Paired analysis of final prevaccination and first postvaccination samples also demonstrated increased spike-specific CD8^+^ T cells postvaccination (*P* = 0.0073) (Fig. 1*I, i*). Conversely, frequency of ORF1a/N-specific CD8^+^ T cells (Fig. 1 *I, ii* and *SI Appendix*, Fig. S2*E*) and spike-specific CD4^+^ T cells did not change with vaccination (Fig. 1*J* and *SI Appendix*, Fig. S2*F*). Influenza tetramers (A2/M1_58,_ A24/PB1_498_, and B35/NP_418_) were included to assess bystander CD8^+^ T cell activation in people with long COVID. Frequencies of these CD8^+^ T cells were stable across two years post SARS-CoV-2 infection, with no difference between people with long COVID and non-LC controls (*SI Appendix*, Fig. S2*G*).

Overall, both people with long COVID and non-LC controls had prototypical robust T cell populations of SARS-CoV-2 epitope-specific CD8^+^ and CD4^+^ T cells following acute infection, convalescence, and vaccination across a two-year time period.

### People with Long COVID Establish and Maintain Large Expansions of SARS-CoV-2 T Cell Responses, Further Boosted Following COVID-19 Vaccination.

**Fig. 2.**
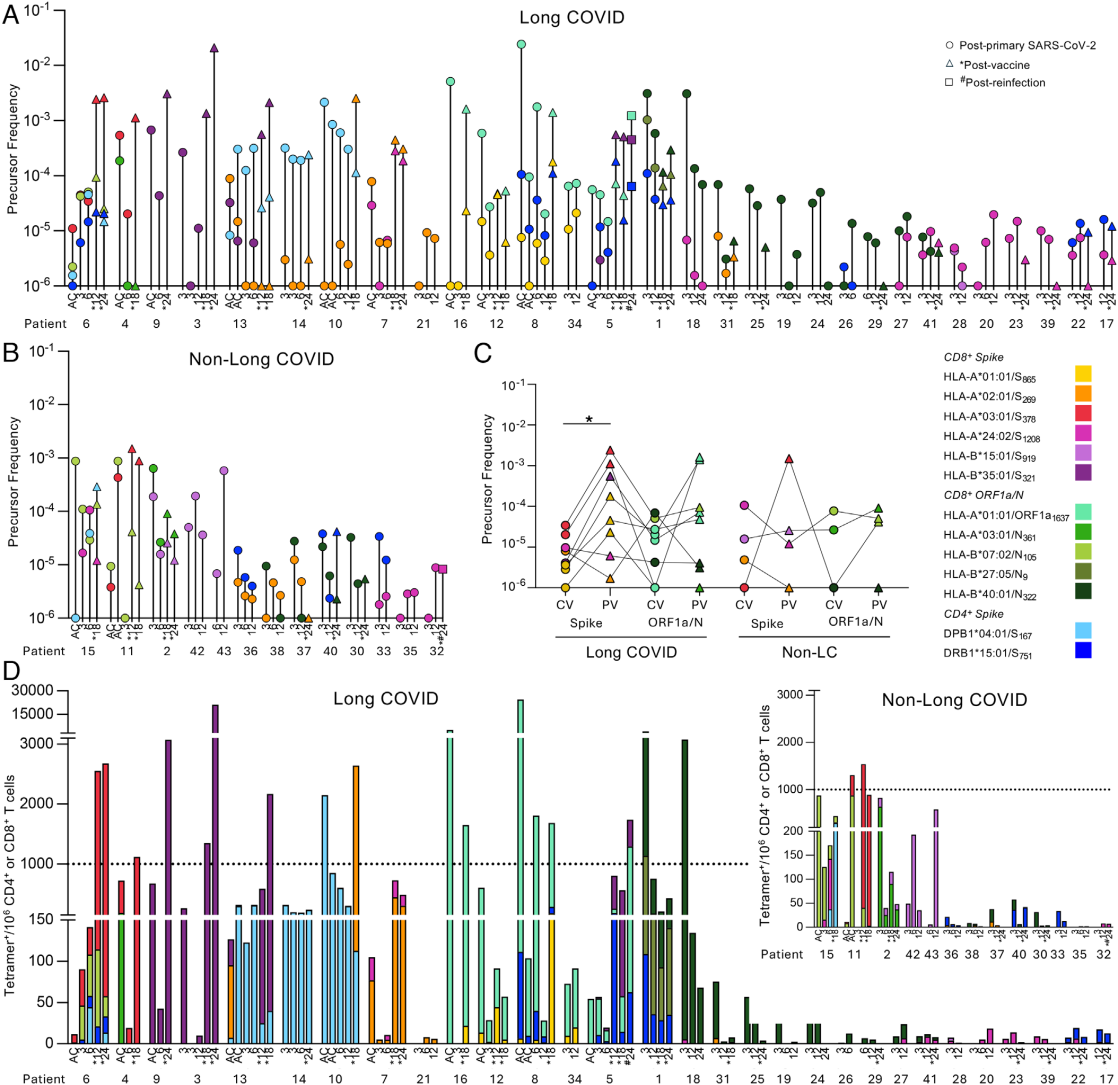
SARS-CoV-2 epitope hierarchy pre- and post-COVID-19 vaccination in people with long COVID. SARS-CoV-2 epitope-specific T cells in longitudinal individuals (>3 mo) graphed per individual per timepoint showing frequencies in (*A*) long COVID and (*B*) non-LC individuals. Plots are colored by epitope. (*C*) Paired S- and ORF1a/N-specific T cell frequencies in people with long COVID and non-LC controls pre- and post-COVID-19 vaccination. Statistical significance determined by the Wilcoxon matched-pairs sign-rank test comparing pre- and post-vaccination. (*A–C*) Frequency of tetramer^+^ cells has been shifted up by 10^−6^ (i.e. no detected tetramer^+^ events displayed as 10^−6^) for visibility on the logarithmic y-axis. (*D*) SARS-CoV-2 tetramer^+^ cells per 10^6^ CD4^+^ or CD8^+^ T cells.

Individual’s SARS-CoV-2-specific T cell frequencies were next assessed as total epitope-specific T cell responses within each individual over time. Based on HLA-I and HLA-II profiles, some individuals had two to four T cell specificities measured with combinations of up to two spike or nonspike CD8^+^ T cell specificities and two spike CD4^+^ T cell specificities (N = 20 long COVID, 8 non-LC), while some individuals only had one T cell specificity assessed (N = 10 long COVID, 5 non-LC). In those with multiple T cell specificities, we assessed immunodominance hierarchical patterns at acute, convalescent, and postvaccination timepoints to determine whether immunodominance hierarchies and/or magnitude changed over time or were impacted by vaccination or reinfection (Fig. 2 *A* and *B*). In people with long COVID, the nonspike CD8^+^ T cell response was typically dominant over spike-specific CD8^+^ T cells at acute and up to three months after infection (epitopes restricted to different HLA allotypes). However postvaccination, nonspike-specific CD8^+^ T cells were relatively stable in frequency, while spike-specific CD8^+^ T cell responses either increased compared to their nonspike counterparts or were further boosted, in agreeance with our earlier grouped analyses of tetramer^+^ frequencies (Fig. 1).

When we analyzed people with long COVID (*N* = 8) pre- and postvaccination with paired spike and nonspike CD8^+^ T cell responses, albeit restricted to different HLA allotypes, nonspike CD8^+^ T cell responses were higher than spike responses prevaccination for 4/8 individuals. However, postvaccination, spike-specific CD8^+^ T cell responses increased or remained stable in 6/8 individuals with long COVID (*P* = 0.0391), while their paired nonspike CD8^+^ T cell responses remained relatively stable in 5/8 individuals (Fig. 2*C*). However, for non-LC controls, only 1/4 individuals showed an increase in spike-specific CD8^+^ T cells, whereas the other three donors had relatively stable frequencies of spike and nonspike T cells.

CD8^+^ T cells from four people with long COVID were assessed longitudinally for HLA-A*01:01-restricted specificities recognizing either ORF1a_1637_ or S_865_ (Fig. 2*A* and *SI Appendix*, Fig. S2*H, i*). Interestingly, A1/ORF1a_1637_-specific T cells were dominant over the 18-mo time period post SARS-CoV-2 infection in both vaccinated (*N* = 3) and nonvaccinated (*N* = 1) individuals. Unfortunately, there was no HLA-A*01:01 expression in non-LC individuals to assess this pattern. Similarly, across different HLA-restrictions, B40/N_322_^+^CD8^+^ T cells, in combination with A2/S_269_, A24/S_1208_ or B27/N_9_ specificities, were generally immunodominant and often even higher in frequency than vaccine-induced spike-specific CD8^+^ T cells (*N* = 4/5) (*SI Appendix*, Fig. S2*H, ii*). However, A2/S_269_^+^CD8^+^ T cell responses in the two non-LC individuals were immunodominant at convalescence 6 to 12 mo over B40/N_322_, although no acute samples were available to determine response kinetics.

One individual with long COVID (donor#5) was vaccinated (BNT162b2) before 12 mo and reinfected by 24 mo post primary SARS-CoV-2 infection (Fig. 2*A* and *SI Appendix*, Fig. S2*H, iii*). At 8 d post primary infection, CD8^+^ T cells were predominantly directed at the A1/ORF1a_1637_ epitope, with an absence of B35/S_321_^+^CD8^+^ and DR15/S_751_^+^CD4^+^ T responses. A1/ORF1a_1637_ was still dominant and stable at 3 (day 109) and 6 mo (day 209), with the emergence of subdominant B35/S_321_^+^CD8^+^ and DR15/S_751_^+^CD4^+^ T cell responses. Post vaccination (day 334-dose 1, 417-dose 2), at 12 and 18 mo sampling (407 and 603 d), T cells directed at both B35/S_321_ and DR15/S_751_ epitopes were higher than those toward A1/ORF1a_1637_ (2.6 to 11.6-fold higher), however postinfection (day 643) by 24 mo (743 d), spike-specific CD8^+^ and CD4^+^ T cells remained at high levels, while A1/ORF1a_1637_-specific CD8^+^ T cells increased to become immunodominant (Fig. 2*A*).

Of 30 individuals with long COVID assessed longitudinally using our personalized tetramer panel, four individuals had very high frequencies of SARS-CoV-2-specific CD8^+^ T cells, over 1000 tetramer^+^ cells per million CD8^+^ T cells (>10^−3^ frequency), early post infection (acute and 3 mo), which was generally attributed to one dominant epitope (A1/ORF1a_1637_ *N* = 2, B40/N_322_ *N* = 2) rather than the sum of multiple T cell specificities (Fig. 2*D*). One individual had very high DPB4/S_167_^+^ CD4^+^ T cells early post infection. Post vaccination, five additional donors reached a similarly >10^−3^ high frequency, and the reinfected individual based on their boosted spike-specific CD8^+^ T cell responses. Conversely, only one non-LC individual (out of 13) reached the threshold frequency of >10^−3^ at acute timepoints (B7/N_105_ and A3/S_378_), which declined at 3 mo postinfection, but re-emerged at high levels following vaccination at 12 to 18 mo due to a vaccine-induced A3/S_378_ response.

Collectively, we show that people with long COVID can establish and maintain large expansions of SARS-CoV-2-specific T cell populations, which can be further boosted by COVID-19 vaccination and reinfection.

### Epitope-Specific T Cell Differentiation and Activation are Driven by Infection and Vaccination Rather than Long COVID.

**Fig. 3.**
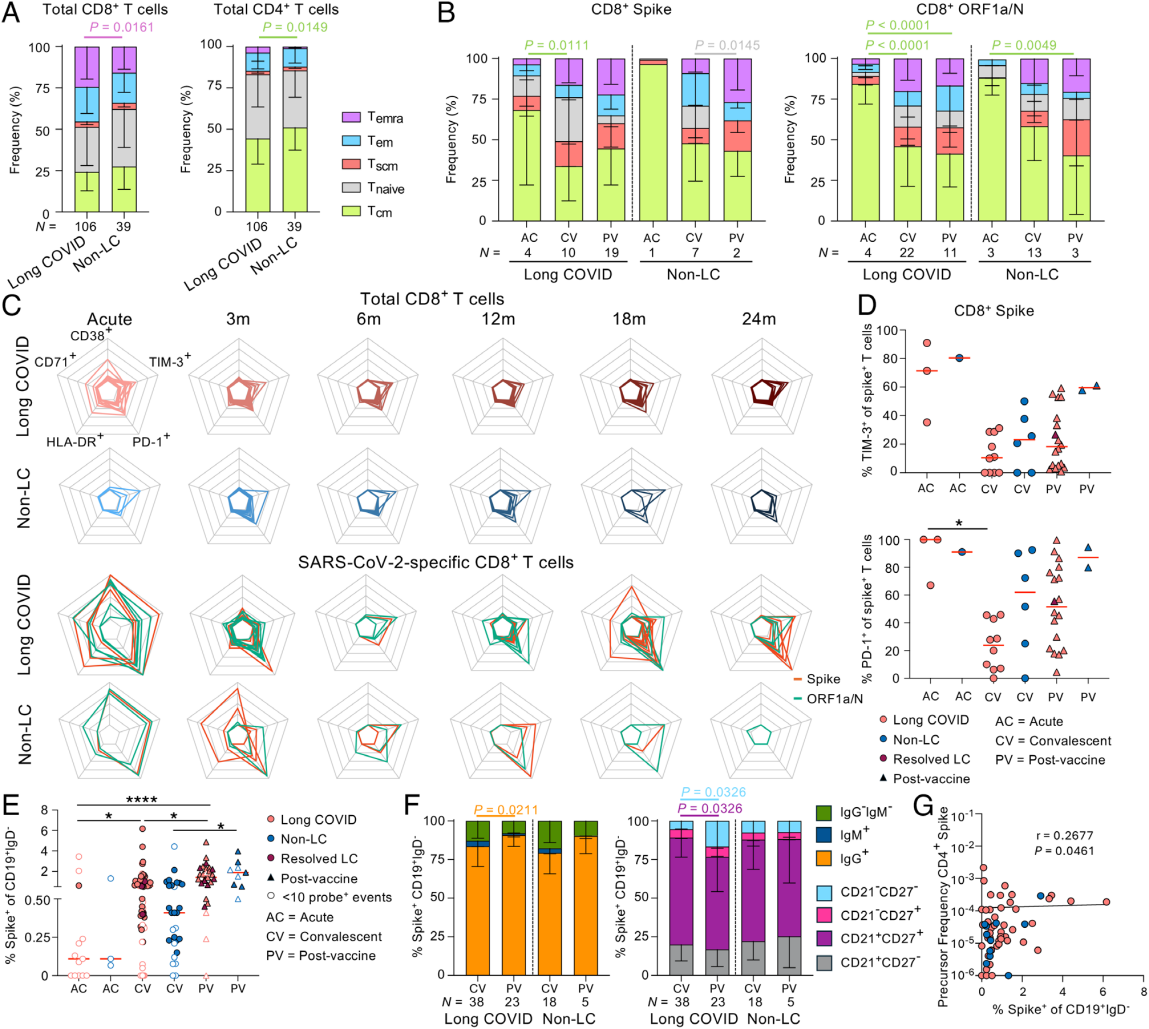
Phenotypic analysis of SARS-CoV-2-specific T and B cells in people with long COVID. Phenotypic profiles split by long COVID status in (*A*) total unenriched CD8^+^ and CD4^+^ T cells, (*B*) enriched CD8^+^ spike-specific and ORF1a/N-specific tetramer^+^ T cells. Seven datapoints are derived from our previous COVID-19 adult cohort (39). (*C*) Radar plots of activation markers (HLA-DR^+^, CD71^+^, CD38^+^, TIM-3^+^, PD-1^+^) of total CD8^+^ T cells and SARS-CoV-2 tetramer^+^ CD8^+^ T cells in long COVID and non-LC groups. Radar shows frequency of expression from 0% (center) to 100% (outer edge). (*D*) TIM-3 and PD-1 expression of spike-specific CD8^+^ T cells in long COVID and non-LC groups. (*E*) Frequency and (*F*) phenotype of SARS-CoV-2 spike-specific probe^+^ B cells in long COVID and non-LC individuals. Samples with <10 probe^+^ events are shown as open symbols. (*G*) Correlation of S-specific CD4^+^ T cells and B cells. Statistical significance determined by (*A, B,* and *F*) Tukey’s multiple comparisons test, (*D* and *E*) Dunn’s multiple comparisons test, and (*G*) Spearman correlation. (*A–D* and *F*) Samples with 10 or more tetramer^+^ or probe^+^ events are included in the phenotypic analysis.

Previous studies showed phenotypic changes in bulk T cell populations in long COVID and during peptide stimulation (26–30). We investigated how ex vivo phenotype and activation of SARS-CoV-2 tetramer-specific T cells changed over time in our long COVID cohort, in comparison to non-LC controls, and how COVID-19 vaccination affected memory and activation phenotypes. Total CD8^+^ and CD4^+^ T cell populations of Tnaive, Tcm, Tem, Temra, and Tscm subsets did not significantly change from acute timepoint to 3-, 6-, 12-, 18-, and 24-mo timepoints for long COVID and non-LC groups (*SI Appendix*, Fig. S3*A*). However, there was a skewing toward terminally differentiated CD8^+^ Temra cells in long COVID people, compared to non-LC controls when all timepoints were pooled together (*P* = 0.0161) (Fig. 3*A*). For spike and nonspike CD8^+^ T cell specificities, the acute timepoint was predominantly of Tcm phenotype (>65%) before decreasing to 30 to 50% at convalescent timepoints in people with long COVID (spike *P* = 0.0111, nonspike *P* < 0.0001) (Fig. 3*B*). These convalescent spike and nonspike CD8^+^ T cell phenotypes in both long COVID and non-LC remained predominantly unchanged from 3 mo to 24 mo (*SI Appendix*, Fig. S3*B*) or following vaccination (Fig. 3*B*). CD4^+^ T cells directed at spike specificities (DPB4/S_167_ and DR15/S_751_) were both of Tem and Tcm phenotypes at acute timepoints for people with long COVID (*SI Appendix*, Fig. S3*C*). However, spike-specific CD4^+^ T cells were mainly of the Tcm phenotype, stable at 3 mo postinfection up to 24 mo or postvaccination (*SI Appendix*, Fig. S3*C*), which was the case for both long COVID and non-LC, and influenza-specific CD8^+^ T cells (*SI Appendix*, Fig. S3*D*). Phenotypes could not be assessed for non-LC CD4^+^ T cell specificities or influenza-specific CD8^+^ T cell specificities at acute timepoints given the low tetramer counts. In cases when epitopes were shared between long COVID and non-LC groups, phenotype distributions across different SARS-CoV-2 CD8^+^ and CD4^+^ T cell specificities were comparable between long COVID and non-LC groups (*SI Appendix*, Fig. S4*A*).

People with long COVID with mild acute disease can have increased expression of T cell inhibitory markers PD-1 and TIM-3 at 3 and 8 mo, with resolution by 24 mo post primary COVID-19 (30, 32). High and prolonged coexpression of CD38 and HLA-DR on T cells has been linked to fatal H7N9 influenza virus infection (45). It is also highly coexpressed on innate and adaptive T cells in severe COVID-19 patients requiring intensive care compared to milder to moderate hospital ward COVID-19 patients (46). To determine activation status of total T cells and SARS-CoV-2 tetramer-specific T cells, we assessed activation markers CD38, HLA-DR, and proliferative marker CD71, as well as TIM-3 and PD-1, linked to T cell exhaustion in chronic diseases and cancers (47). We observed high expression of all 5 markers in SARS-CoV-2-specific CD8^+^ and CD4^+^ T cells and moderate expression in total CD8^+^ and CD4^+^ T cells during acute SARS-CoV-2 infection, in both long COVID and non-LC groups (Fig. 3*C* and *SI Appendix*, Fig. S4*B*). PD-1 remained highly expressed in SARS-CoV-2-specific T cells at 3 mo, but was reduced by 6 mo (Fig. 3*C*), with significantly lower PD-1 expression at convalescence compared to acute disease in patients with long COVID (CD8^+^ spike *P* = 0.0295, CD8^+^ ORF1a/N *P* = 0.0348, CD4^+^ spike *P* = 0.0157) (Fig. 3*D* and *SI Appendix*, Fig S4*C*). Although there was a trend toward increased PD-1 and TIM-3 expression levels following vaccination, this was not significant on spike-specific CD8^+^ or CD4^+^ T cells (Fig. 3*D* and *SI Appendix*, Fig. S4*C*). Conversely, expression of CD38, HLA-DR, and CD71 was minimal at all convalescent time-points, pre- and postvaccination, in long COVID or non-LC groups. Thus, we did not observe prolonged activation of SARS-CoV-2 CD8^+^ or CD4^+^ T cells (or total T cells) in people with long COVID up to 2 years postinfection.

The frequency of bulk CD19^+^ B cells, spike-/N-specific CD19^+^IgD^−^ memory B cells was reported unaltered in long COVID stemming from mild acute disease (32), thus we assessed antigen-specific B cells using spike and N fluorescent probes in our cohort of people with long COVID and non-LC controls (*SI Appendix*, Fig. S1*B**)*. Circulating spike- and N-specific CD19^+^IgD^−^ B cells during acute infection (day 0 to 22) were low in both long COVID and non-LC individuals. Spike-specific B cells significantly increased by convalescence in people with long COVID (*P* = 0.0333), with a similar trend observed in N-specific B cells and non-LC controls (Fig. 3*E* and *SI Appendix*, Fig. S5*A*). Following resolution of acute disease, N-specific B cell frequencies remained stable over the 24 mo (*SI Appendix*, Fig. S5*B*), while spike-specific B cells increased significantly at 18 and 24 mo postinfection as a result of vaccination (*SI Appendix*, Fig. S5*C*). Frequencies of spike-specific B cells increased in both people with long COVID and non-LC between vaccine-naive convalescence and postvaccination (LC *P* = 0.0199, non-LC *P* = 0.0104) (Fig. 3*E* and *SI Appendix*, Fig. S5*A*). People with long COVID had increased frequency of IgG^+^ spike-specific B cells postvaccination (*P* = 0.0211), with a similar trend observed in non-LC, (Fig. 3 *F*, *Left*). Additionally, an increase in CD21^−^CD27^−^ B cells (*P* = 0.0326) and decrease in CD21^+^CD27^+^ resting B cells (*P* = 0.0326) was found in people with long COVID (Fig. 3 *F*, *Right*). Meanwhile, N-specific B cell isotype and phenotype were not affected by vaccination (*SI Appendix*, Fig. S5 *D* and *E*). The changes in spike-specific memory B cell isotype (*SI Appendix*, Fig. S5*F*) and phenotype (*SI Appendix*, Fig. S5*G*) were also observed when data were graphed against time, with all but two of the people with long COVID vaccinated by their final study visit. Finally, the frequency of spike-specific CD4^+^ T cells weakly correlated with the spike-specific B cells when long COVID and non-LC were combined (r = 0.2677, *P* = 0.0461) (Fig. 3*G*).

Overall, we show that SARS-CoV-2 antigen-specific T cells and B cells in people with long COVID have prototypical memory and activation phenotypes following resolution of acute infection and post-COVID-19 vaccination.

### People with Long COVID Display Prominent SARS-CoV-2-Specific T Cell Receptor Signatures Overlapping with Non-LC TCRs during Infection and Vaccination.

Antigen-specific T cell responses are shaped by their TCRαβ repertoires, encoded by the V(D)J gene segment usage and CDR regions. Here, we defined TCR signatures of SARS-CoV-2-specific CD8^+^ and CD4^+^ T cells in long COVID and fully recovered individuals over time. We assessed TCR sequences across six SARS-CoV-2 specificities: A2/S_269,_ A24/S_1208_, A3/S_378,_ B7/N_105_, B35/S_321_, and DPB4/S_167_. No TCR data currently exist for A3/S_378_ and B35/S_321_ epitopes.

**Fig. 4.**
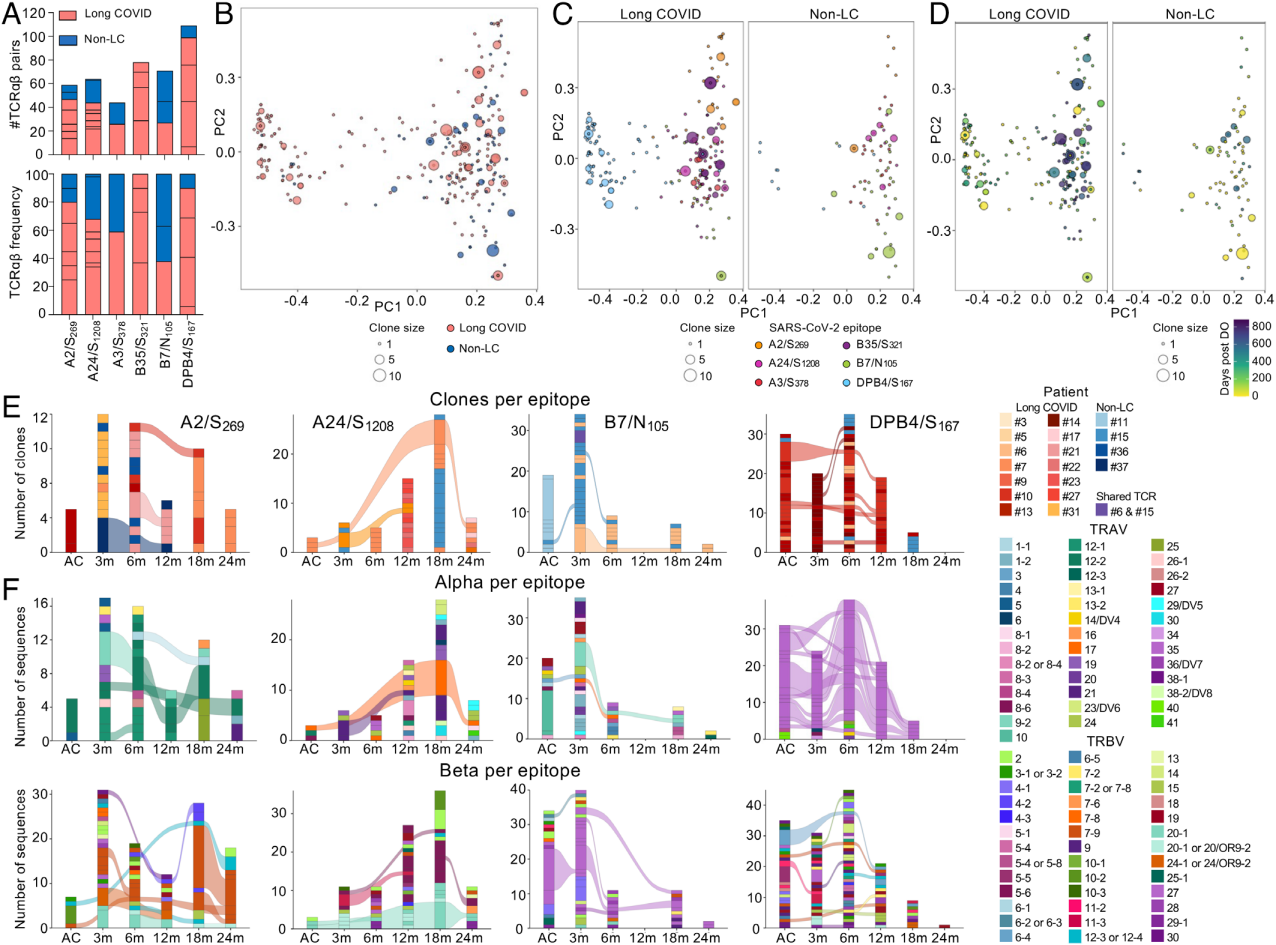
SARS-CoV-2-specific T cell receptor repertoires of people with long COVID overlap with non-LC individuals. (*A*) Number and frequency of TCR clonotypes sequenced per SARS-CoV-2 epitope. Bar segments indicate the contributions from individual donors. (*B*) kPCA plot of long COVID and non-LC TCR clonotypes together by clonotype size. (*C*) kPCA plots of long COVID and non-LC TCR clonotypes colored by epitope or (*D*) by sampling timepoint as days post disease onset. (*E*) Combined long COVID and non-LC alluvial plots of A2/S_269,_ A24/S_1208_, B7/N_105_, and DPB4/S_167_ TCR repertoires, showing sharing of TCRαβ clones across the time-points by the band connections in colors representing individuals. (*F*) Alluvial plots showing sharing of TCRα and TCRβ CDR3 chains across the time-points by the band connections in colors representing TRAV and TRBV usage.

To assess TCRαβ repertoires, we single-cell sorted and sequenced 424 SARS-CoV-2-specific tetramer^+^ T cells from 16 people with long COVID and four non-LC controls at acute, convalescent, and postvaccination timepoints. Distribution of TCR sequences per epitope was spread between long COVID (38 to 80%, *N* = 1 to 6 per epitope) and non-LC individuals (*N* = 1 to 2 per epitope) with two exceptions (Fig. 4*A*); B35/S_321_ TCR sequences all came from people with long COVID (*N* = 4; no non-LC in this cohort expressed HLA-B*35:01) and DPB4/S_167_ TCR sequences were 91% from people with long COVID. kPCA analyses generated by TCRdist revealed that TCR clonotypes from people with long COVID were evenly interspersed with non-LC TCR clonotypes (Fig. 4*B*). Both long COVID and non-LC groups showed two separate clusters, mainly based on the separation of CD8^+^ and CD4^+^ T cell epitopes (Fig. 4*C*) rather than timing of sampling (Fig. 4*D*). In both groups, spike-specific CD8^+^ TCR clonotypes mainly projected upward in the kPCA plot while B7/N_105_ TCR clonotypes and their clonal expansions projected downward away from the spike CD8^+^ TCR clonotypes.

The A2/S_269_ TCR repertoire is widely studied with known prominent CDR3α motifs (TRAV12-2 _ TRAJ30 “CAVNXXDKIIF” and TRAV12-1 _ TRAJ43 “CVVNXXDDMRF,” where “X” denotes any amino acid) and CDR3β motifs (TRBV7-9 “CASSPDIEQYF” and TRBJ2-2 “CAS-NTGELFF”) (39, 40, 48). Similar A2/S_269_ motifs were present in long COVID and non-LC individuals (*SI Appendix*, Table S2). TRAV12-2 _ TRAJ30 CAVNXDDKIIF was found in 3 people with long COVID from 3 mo up to 24 mo, 2 at convalescence and 1 at postvaccination timepoints. These TCRα chains were either paired with TRBV7-9 (CASSPDIVQFF, CASSSDIXAFF, CASSPDIKQYF) or TRBV20-1 _ TRBJ2-2 clonotypes (CSARDHQAQNTGELFF). The other prominent TRAV12-1 _ TRAJ43 CVVNXXXDMRF motif was also present in 3 long COVID and 1 non-LC individuals at convalescent timepoints with different TCRβ chains (*SI Appendix*, Table S2).

TCR repertoires against the A24/S_1208_ and B7/N_105_ epitopes showed expanded clonotypes within long COVID and non-LC individuals (>1 clonotype/timepoint). However, most of these clonotypes were private, not shared between donors in either group, with the exception of one B7/N_105_ clonotype in long COVID donor#6 (2 sequences) and non-LC #15 (1 sequence) (Fig. 4*E* and *SI Appendix*, Table S2). A3/S_378_ TCR repertoire was less expanded but highly diverse in one individual with long COVID and one non-LC individual. B35/S_321_ TCR repertoire was driven by large clonal expansions of 1 to 2 private clonotypes in individuals with long COVID at convalescent timepoints (*N* = 4) (*SI Appendix*, Table S2). Although clonally expanded within donors, the high diversity between donors was previously observed in A24/S_1208_ and B7/N_105_ TCR repertoires from COVID-19 individuals (39, 40, 49).

DPB4/S_167_ TCR repertoire included the highly prominent TRAV35_TRAJ42 CAXXNYGGSQGNLIF TCRα motif representing 73% of total DPB4/S_167_ clonotypes (68/93 alpha sequences), observed in all six sequenced donors, including five people with long COVID and one non-LC (*SI Appendix*, Table S2). These TCRα chains were paired with diverse TCRβ chains and were clonally expanded within donors at acute, convalescent, and postvaccination timepoints; however, the TCRβ chains were not shared between donors. The heavily biased TRAV35_TRAJ42 CAXXNYGGSQGNLIF TCRα motif being paired with different TCRβ chains was observed in SARS-CoV-2 infection and vaccination cohorts (40–42).

Given the overlap in SARS-CoV-2-specific TCRαβ repertoires between long COVID and non-LC groups, we compiled all individuals to generate TCRαβ, TCRα and TCRβ alluvial plots per epitope to assess whether the same clonotype or TCRα or TCRβ sequence was observed within individuals at multiple timepoints up to 24 mo (Fig. 4 *E* and *F* and *SI Appendix*, Fig. S6). Indeed, we observed a high level of sharing over time within individuals for the TCRα chain (*N* = 14) and the TCRβ chain (*N* = 16) across different epitopes, even following vaccination, which consisted of prominent TCRα/β motifs, and sequences from private and expanded clonotypes (Fig. 4*F**)*. Finally, 13 CD8^+^ and 7 CD4^+^ TCRαβ clones were identified across timepoints within individuals, providing strong evidence of clonal persistence over time in individuals with and without long COVID (Fig. 4*E* and *SI Appendix*, Table S2).

Thus, SARS-CoV-2-specific CD4^+^ and CD8^+^ T cells from long COVID and non-LC individuals are similar in nature. Both groups display key TCR signatures that can be detected over 2 y following disease onset.

## Discussion

Even in the era of COVID-19 vaccination, a small but important minority of individuals experience long COVID, reducing quality of life and contributing to health-care burden (18). Multiple clinical syndromes occur during long COVID, some of which are debilitating. Possible mechanisms of long COVID are still far from clear, although immune perturbations including inflammatory mediators, antibodies, and total T cell responses were postulated (19, 30, 31). As T cell responses at the SARS-CoV-2 epitope level, together with their TCR signatures, have not been explored directly ex vivo in long COVID, we investigated establishment and long-term persistence of SARS-CoV-2 epitope-specific T cells within circulating blood in people with long COVID. Our data provide evidence that SARS-CoV-2 peptide–HLA tetramer-specific CD4^+^ and CD8^+^ T cells, together with probe-specific B cells, are established in people with long COVID, and maintained over 24 mo, similarly to SARS-CoV-2-specific T cells and B cells detected in non-long COVID individuals. Our study defines in-depth ex vivo magnitude, phenotype and clonal TCRαβ signatures of SARS-CoV-2-specific CD8^+^ and CD4^+^ T cells in people with long COVID across 2 y. As the recall of memory T cells promotes rapid recovery and ameliorates disease outcomes, establishment of robust memory T cells is of key importance for protection against subsequent infections, especially when the virus mutates. Our data provide evidence for the establishment and persistence of prototypical and robust SARS-CoV-2 antigen-specific T cell memory populations in people with long COVID at 2 y after SARS-CoV-2 infection, indicating that individuals who recovered from long COVID are equipped with T cell memory pools in a similar way to non-long COVID individuals. This observation was unaffected by the burden or severity of clinical syndromes in long COVID.

Robust effector CD8^+^ T cell responses are associated with superior viral control and milder outcomes, including influenza and COVID-19 (24, 25, 50). T cells also establish long-term memory populations and, as they are directed toward epitopes encompassing conserved virus-derived peptides, T cells recognize a broad range of viral variants (40) and thus provide protection when new viral variants emerge (51). In long COVID, T cells were previously assessed at the bulk T cell population level or following stimulation with overlapping SARS-CoV-2 peptides (26–30), thus our study contributes substantially to the literature by providing in-depth understanding of the magnitude, phenotypic, and clonotypic features of circulating SARS-CoV-2 epitope-specific CD4^+^ and CD8^+^ T cell responses in long COVID directly ex vivo. Using 13 SARS-CoV-2 peptide–HLA tetramers detecting immunodominant SARS-CoV-2 T cell specificities, we tracked SARS-CoV-2-specific CD8^+^ and CD4^+^ T cells in individuals from their first SARS-CoV-2 infection, through their primary vaccination, over 24 mo. Frequencies of ORF1a- and nucleocapsid-specific T cells remained stable over 24 mo, indicating long-term persistence of SARS-CoV-2-specific memory pools in long COVID. SARS-CoV-2-specific T cells from individuals with long COVID were also enriched for the central memory phenotype. We and others previously compared ex vivo SARS-CoV-2-specific CD8^+^ and CD4^+^ T cell responses and their TCR repertoires, using peptide/HLA-tetramer enrichment techniques in blood of SARS-CoV-2-infected children and adults (38–40, 49), blood and tissues of prepandemic children and adults (38, 39) and following COVID-19 mRNA vaccination versus infection (42, 44). Adults who have recovered from COVID-19 have stable central memory CD4^+^ and CD8^+^ T cells directed toward a range of SARS-CoV-2 spike and nonspike epitopes. Conversely, COVID-19 convalescent children display more stem-cell-like phenotype, while prepandemic responses are largely naïve (40). These findings provide evidence that individuals with long COVID can generate SARS-CoV-2-specific T cell memory pools of the optimal central memory phenotype similar to adult SARS-CoV-2 infected patients who fully recover.

COVID-19 vaccination with both BNT162b2 or ChAdOx1 induces robust CD4^+^ and CD8^+^ tetramer^+^ T cells toward immunodominant spike-specific epitopes in healthy participants (42, 44) and high-risk groups, including hematology patients, individuals with autoimmunity, and First Nations people (41, 52, 53). Importantly, patients lacking SARS-CoV-2-specific IgG antibodies could still generate T cell responses, with similar ex vivo tetramer^+^ T cell frequencies to patients with RBD-specific IgG antibodies and healthy individuals (41, 53). Here, we demonstrate that spike-specific CD8^+^ and CD4^+^ T cells were boosted by SARS-CoV-2 vaccination, indicating beneficial effects of COVID-19 vaccination in individuals with or recovered from long COVID. Although immunization in both fully recovered and people with long COVID altered immunodominance hierarchy of SARS-CoV-2 epitopes, influenza-specific CD8^+^ T cells were stable across 24 mo, suggesting no bystander-activation of CD8^+^ T cells directed against other respiratory viruses.

We also investigated ex vivo CD4^+^ and CD8^+^ TCR repertoires toward SARS-CoV-2 epitopes across 2 y postinfection to define antigen-specific T cells in long COVID. Importantly, TCRαβ repertoire composition and diversity were established and maintained throughout long COVID, vaccination to 2 y postinfection. Previous findings showed that following both SARS-CoV-2 infection and vaccination, SARS-CoV-2-specific TCR sequences reveal diverse repertoires characterized by prominent TCR motifs shared between different individuals (39, 40, 42, 44, 48, 49). We observed known prominent motifs specific for A2/S_269_ and DPB4/S_167_ in people with long COVID, while diverse but expanded repertoires were evident for A24/S_1208_ and B7/N_105_, echoing previous findings early in the pandemic (39, 40, 42, 48, 49)_._ We show similarities between SARS-CoV-2-specific CD4^+^ and CD8^+^ T cells from people with long COVID and non-LC controls, with both groups displaying key TCR signatures and TCRαβ clonotypes that can be detected over 2 y following disease onset, suggesting that, at least at the clonotype level, T cell responses are unaffected by long COVID.

Overall, our T cell studies defined ex vivo SARS-CoV-2-specific T cells at quantitative, phenotypic, and clonotypic levels to understand primary and recall responses, providing key insights into CD8^+^ and CD4^+^ T cell responses in fully recovered and people with long COVID.

## Materials and Methods

### Study Participants and Ethics Statement.

Hospitalized COVID-19 patients were recruited via Sentinel Travelers Research Preparedness Platform for Emerging Infectious Diseases (SETREP-ID) Study at sites in Melbourne, Australia. Patients were enrolled during 2020 COVID-19 pandemic wave (January–September 2020), followed longitudinally with sample and data collection to 6 mo. A subset (*N* = 19) reconsented to additional follow-up to 2 y, with study visits at ~6-monthly intervals. RT-PCR-positive COVID-19 patients were also recruited from Prince of Wales Hospital during quarantine in Hong Kong between February and October 2020, longitudinally sampled at ~6-mo intervals. Long COVID specific-symptom data were collected during follow-up visits.

SETREP-ID study was approved by Melbourne Health (HREC/17/MH/53) and University of Melbourne (#1749349, #13344) human research ethics committees (HREC). The Hong Kong COVID-19 patient cohort was approved by institutional review board HKU/HA Hong Kong West Cluster (UW 20-273, UW20-169, UW 20-132), Joint Chinese University of Hong Kong-New Territories East Cluster Clinical Research Ethics Committee (CREC 2020.229) and University of Melbourne HREC (#25684). Cohort summary demographics are described in *SI Appendix*, Table S1. The study was conducted in compliance with conditions of the ethics committee approval, NHMRC National Statement on Ethical Conduct in Human Research 2007 (updated 2018) and Note for Guidance on Good Clinical Practice (CPMP/ICH-135/95).

Informed consent was obtained from all participants. Cohort clinical details, ex vivo tetramer enrichment, B cell probe staining, and TCR analysis are described in *SI Appendix*.

## Supplementary Material

Appendix 01 (PDF)

Dataset S01 (XLSX)

## Data Availability

All study data are included in the article. The TCR sequences in this study were uploaded to Mendeley Data under the following access code: https://doi.org/10.17632/ffw3jrbdcb.1 (54).
