# Supplementary material for: SARS-CoV-2-specific CD8+ T cells from people with long COVID establish and maintain effector phenotype and key TCR signatures over 2 years
Source: Proc Natl Acad Sci U S A. 2024 Sep 16;121(39):e2411428121. doi: 10.1073/pnas.2411428121 (PMC11441481; doi:10.1073/pnas.2411428121)
Supplement: Supplementary file 1 — Appendix 01 (PDF) [file pnas.2411428121.sapp.pdf]

*SI Appendix*

Supplementary Information Text

## METHODS

**People with long COVID and non-LC controls.** Individuals disease severity during ac SARS-CoV-2 infection was designed in accordance with the NIH COVID-19 Treatment Guidelines Panel (1). SETREP-ID data from Melbourne hospitals were collected and managed using REDCap electronic data capture tools hosted at The University of Melbourne. Methods for blood collection, PBMC isolation and HLA typing have previously been described for Melbourne (2) and Hong Kong (3).

Long COVID was defined as persistent symptoms at least 3 months after infection according to common case definitions (4). Self-reported symptoms during follow-up were classified into clinical syndromes as follows: neurological (including fatigue, headache, memory issues, mental fog, lack of concentration, mental confusion, dizziness, tremor, paraesthesia, loss of taste or smell, problems speaking, balance issues), respiratory (including cough, dyspnoea, pain on breathing), coryzal (sputum, cold symptoms, dry cough) haematological (anaemia), vascular (swollen ankles), musculo-skeletal (joint pain, muscle pain, muscle aches, muscle weakness), gastro-intestinal (diarrhoea, nausea, vomiting, abdominal pain, constipation), dermatological (skin rashes, skin lumps), loss of appetite, Reynaud's Syndrome, chest pain, problems sleeping, weight changes and menstrual changes. For analytical purposes, these were collapsed into 6 clinical syndromes – neurological, cardio-respiratory, psychological, musculoskeletal, gastrointestinal and dermatological.

**Longitudinal SARS-CoV-2-specific T and B cell responses.** SARS-CoV-2 epitope-specific $CD8^+$ and $CD4^+$ T cell responses were assessed using 1-10x$10^6$ cryopreserved PBMCs, thawed and stained with fluorophore-conjugated HLA class I and II tetramers (Prof Jamie Rossjohn, Monash University) representing common SARS-CoV-2 epitopes. These included HLA-A*01:01/ORF1a$_{1637}$ (TTDPSFLGRY), HLA-A*01:01/S$_{865}$ (LTDEMIAQY), HLA-A*02:01/S$_{269}$ (YLQPRTFLL), HLA-A*03:01/N$_{361}$ (KTFPPTEPK), HLA-A*03:01/S$_{378}$ (KCYGVSPTK), HLA-A*24:02/S$_{1208}$ (QYIKWPWYI), HLA-B*07:02/N$_{105}$ (SPRWYFYYL), HLA-B*15:01/S$_{919}$ (NQKLIANQF), HLA-B*27:05/N$_{9}$ (QRNAPRITF), HLA-B*35:01/S$_{321}$ (QPTESIVRF), HLA-B*40:01/N$_{322}$ (MEVTPSGTWL) and HLA-DPA1*01:03/DPB1*04:01/S$_{167}$ (TFEYVSQPFLMDLE)(2, 5-8). HLA-DRB1*15:01/S$_{751}$ (NLLLQYGSFCTQLNRAL) monomers were sourced from ProImmune. Influenza class I tetramers (Prof Stephanie Gras, La Trobe University), HLA-A*02:01/M1$_{58}$ (GILGFVFTL), HLA-A*24:02/PB1$_{498}$ (RYGFVANF) and 5x HLA-B*35:01/NP$_{418}$ variants (LPFDRTTIM, LPFDKTTIM, LPFEKSTIM, LPFEKSTVM, LPFERATIM), were included for staining where possible. Class I tetramers were conjugated to PE or APC, and class II DPB4/S$_{167}$ and DR15/S$_{751}$ tetramers were conjugated to PE and APC, respectively. After 1 hr of incubation at room temperature in the dark, stained cells were washed and incubated with anti-PE and anti-APC microbeads for 30 mins on ice before performing tetramer-associated magnetic enrichment (TAME) with Miltenyi Biotec LS columns (Bergisch Gladbach, Germany) (2, 6, 9, 10). Unenriched, enriched and flowthrough fractions were cell surface stained with anti-human CD71 BV421 (#562995), CD4 BV650 (#563875), CD27 BV711 (#563167), CD38 BV785 (#563964), LAG-3 BUV395 (#569247), TIM-3 BUV737 (#568680), CD45RA FITC (#555488), CD8 PerCP-Cy5.5 (#565310), CD95 PE-CF594 (#562395), PD-1 PE-Cy7 (#561272), CCR7 AF700 (#561143), CD14 APC-H7 (#560180), CD19 APC-H7 (#560177) (all from BD Biosciences, Franklin Lakes, NJ, USA), CD3 BV510 (#317332, Biolegend, San

Diego, CA, USA), HLA-DR BV605 (#307640, Biolegend), L/D NIR (#L10119, Invitrogen, Waltham, MA, USA). Cells were washed and fixed for acquisition on a BD LSR Fortessa II. For some experiments, enriched tetramer$^+$ CD8$^+$ and CD4$^+$ T cells were index-sorted into single cells on a BD FACSAria III using the software BD FACS DIVA v8.0.1 ribed (2, 6). Data were analyzed using FlowJo v10 software. Tetramer-positive frequencies corresponding to <10 tetramer$^+$ events were not included in phenotypic analysis and displayed as open symbols. T cell activation markers were visualized as radar plots using the fmsb package v.0.7.6 in R v.4.2.1 (11).

Spike-and nucleocapsid protein-specific B cells from the ancestral strain were measured on thawed TAME-flow through fractions. Cells were stained with wildtype Spike recombinant probe conjugated to BV421 and nucleocapsid protein recombinant probe conjugated to PE and APC, as well as anti-human CD3 BV510 (#317332), CD8a BV510 (#301048), CD10 BV510 (#312220), CD14 BV510 (#301842), CD16 BV510 (#302048), CD27 BV605 (#302830), CD71 BV650 (#334116) (all from Biolegend), IgM BUV395 (#563903), CD21 BUV737 (#612788), IgG BV786 (#564230), CD20 AF700 (#560631), IgD PE-Cy7 (#561314), Free-SA BV510 (#563261) (all from BD Biosciences), CD19 ECD (#IM2708U, Beckman Coulter, Indianapolis, IN, USA) and Live Dead AQUA (#L34966, ThermoFisher, Waltham, MA, USA), fixed and acquired on a BD LSRII Fortessa, essentially as described (12, 13).

**TCRαβ analysis.** TCR sequences were generated by amplifying CDR3α and CDR3β regions from single cells using multiplex-nested RT-PCR (2, 14). TCR sequences were analyzed by IMGT/V-QUEST and visualised as alluvial plots using the ggalluvial v.0.12.5 (15) and circlize v.0.4.16 (16) packages in R v.4.2.1 (11). Paired alpha and beta TCR sequences were analysed by tcrdist (17, 18) in Python v.2.7 for determination of pairwise distances between clonotypes.

**Statistical analysis.** Statistical significance of nonparametric datasets (two-tailed) were determined using GraphPad Prism v9 software. Correlation (r), Wilcoxin signed-rank test (paired 2 groups), Kruskal-Wallis test (unmatched) with Dunn's multiple comparisons and Tukey's multiple comparison test were used and indicated in the figure legends. Where multiple comparisons tests have been used, only relevant comparisons have been depicted, i.e. comparisons between timepoints or vaccination status within LC or non-LC groups or between long COVID and non-LC groups within a timepoint/vaccination status. $*P \leq 0.05$, $**P \leq 0.01$, $***P \leq 0.001$, $****\ P \leq 0.0001$.

**Table S1.** Clinical cohort

| | Long COVID | Non-LC |
|---|---|---|
| Number of individuals, *N* | 31 | 19 |
| Age, mean (range) | 52 (19-85) | 49 (20-74) |
| Female, *N* (%) | 19 (61) | 6 (32) |
| Days post SO, mean (range) | | |
| Ac | 9 (2-22) | 7 (0-11) |
| 3 month | 73 (19-122) | 65 (26-119) |
| 6 month | 211 (181-259) | 185 (166-237) |
| 12 month | 364 (278-455) | 360 (270-431) |
| 18 month | 579 (498-628) | 584 (561-599) |
| 24 month | 756 (645-886) | 748 (708-776) |
| Resolved LC by end of study, *N* (%) | 12 (29) | - |
| Vaccinated by end of study, *N* (%) | 21 (68) | 7 (37) |
| COVID vaccine, *N (%)* | | |
| BNT162b2 | 19 (61) | 5 (26) |
| mRNA-1273 | 1 (3) | 0 (0) |
| ChAdOx1 | 4 (13) | 2 (11) |

| | | |
|---|---|---|
| NVX-CoV2373 | 1 (3) | 0 (0) |
| PiCoVacc | 1 (3) | 0 (0) |
| Severity of original infection, *N* (%) | | |
| Asymptomatic | 2 (6) | 1 (5) |
| Mild | 4 (13) | 9 (47) |
| Moderate | 14 (45) | 6 (32) |
| Severe | 5 (16) | 2 (11) |
| Critical | 6 (19) | 1 (5) |
| Long COVID Phenotype, *N* (%) | | |
| Neurological | 28 (90) | - |
| Cardio-Respiratory | 18 (58) | - |
| Psychological | 2 (6) | - |
| Musculoskeletal | 17 (55) | - |
| Gastrointestinal | 10 (32) | - |
| Dermatological | 5 (16) | - |

**Table S2.** Compiled TCR table

| A2/$S_{269}$ | | | | | Donor | 7 | 7 | 7 | 7 | 10 | 10 | 10 | 13 | 13 | 21 | 21 | 36 | 36 | 36 | 37 | 37 | 31 | 31 |
|---|---|---|---|---|---|---|---|---|---|---|---|---|---|---|---|---|---|---|---|---|---|---|---|
| | | | | | Long COVID | Y | Y | Y | Y | Y | Y | Y | Y | Y | Y | Y | N | N | N | N | N | Y | Y |
| | | | | | Timepoint (months) | ac | 3 | 18 | 24 | 6 | 12 | 18 | ac | 6 | 6 | 12 | 3 | 6 | 12 | 3 | 12 | 3 | 12 |
| TRAV | TRAJ | CDR3α | TRBV | TRBJ | CDR3β | | | | | | | | | | | | | | | | | | |
| TRAV-ND | TRAV-ND | TRAV-ND | TRBV12-3/TRBV12-4 | TRBJ2-2 | CASSDANTGELFF | 1 | | 1 | 3 | | | | | | | | | | | | | | |
| TRAV-ND | TRAV-ND | TRAV-ND | TRBV7-9 | TRBJ1-1 | CASSSDIEAFF | 1 | | | 1 | | | | | | | | | | | | | | |
| TRAV26-1 | TRAJ37 | CIVRVLIYGSSNTGKLIF | TRBV27 | TRBJ2-7 | CASSPGTAAYEQYF | | 1 | | | | | | | | | | | | | | | | |
| TRAV-ND | TRAV-ND | TRAV-ND | TRBV4-2 | TRBJ2-3 | CASGPPASGDTDTQYF | | | 2 | | | | | | | | | | | | | | | |
| TRAV-ND | TRAV-ND | TRAV-ND | TRBV7-9 | TRBJ2-1 | CASSPDIVQFF | | | 10 | 6 | | | | | | | | | | | | | | |
| TRAV12-2 | TRAJ30 | CAVNRDDKIIF | TRBV7-9 | TRBJ2-1 | CASSPDIVQFF | | | 4 | | | | | | | | | | | | | | | |
| TRAV16 | TRAJ28 | CALRGHRGAGSYQLTF | TRBV4-2 | TRBJ2-3 | CASSPLLSADTQYF | | | 1 | | | | | | | | | | | | | | | |
| TRAV25 | TRAJ18 | GSTLGRLYF | TRBV15 | TRBJ2-2 | CATQDANTGELFF | | | 2 | | | | | | | | | | | | | | | |
| TRAV9-2 | TRAJ22 | CARDLISSGSARQLTF | TRBV20-1 | TRBJ2-2 | CSARDEWGANTGELFF | | | 1 | | | | | | | | | | | | | | | |
| TRAV-ND | TRAV-ND | TRAV-ND | TRBV20-1/TRBV20/OR9-2 | TRBJ2-2 | CSARDEWGANTGELFF | | | | 1 | | | | | | | | | | | | | | |
| TRAV-ND | TRAV-ND | TRAV-ND | TRBV7-9 | TRBJ1-1 | CASSLDIEAFF | | | | 1 | | | | | | | | | | | | | | |
| TRAV-ND | TRAV-ND | TRAV-ND | TRBV7-9 | TRBJ2-1 | CASSWDIEQFF | | | | 1 | | | 1 | | | | | | | | | | | |
| TRAV1-2 | TRAJ29 | CAVRPPSKNTPLVF | TRBV-ND | TRBV-ND | TRBV-ND | | | | 1 | | | | | | | | | | | | | | |
| TRAV12-2 | TRAJ30 | CAVNEDDKIIF | TRBV7-9 | TRBJ1-1 | CASSSDIEAFF | | | | 1 | | | | | | | | | | | | | | |
| TRAV12-2 | TRAJ30 | CAVNEDDKIIF | TRBV7-9 | TRBJ1-1 | CASSSDIQAFF | | | | 1 | | | | | | | | | | | | | | |
| TRAV21 | TRAJ8 | CAVSSKGFQKLVF | TRBV2 | TRBJ2-2 | CARRTDQNTGELFF | | | | 2 | | | | | | | | | | | | | | |
| TRAV8-3 | TRAJ21 | CAVGPYNFNKFYF | TRBV7-9 | TRBJ1-1 | CASSPDIGAFF | | | | 1 | | | | | | | | | | | | | | |
| TRAV1-1 | TRAJ23 | CAVKGVGGKLIF | TRBV12-3/TRBV12-4 | TRBJ2-2 | CAVEEANTGELFF | | | | | 1 | | 1 | | | | | | | | | | | |
| TRAV12-1 | TRAJ43 | CVVNNNNDMRF | TRBV4-2 | TRBJ2-7 | CASGEANSGEQYF | | | | | 1 | | | | | | | | | | | | | |
| TRAV-ND | TRAV-ND | TRAV-ND | TRBV4-2 | TRBJ2-7 | CASGEANSGEQYF | | | | | | 1 | 2 | | | | | | | | | | | |
| TRAV25 | TRAJ34 | YNTDKLIF | TRBV7-9 | TRBJ2-1 | CASSWDIEQFF | | | | | | | 3 | | | | | | | | | | | |
| TRAV12-2 | TRAJ3 | CAVISDSSASKIIF | TRBV10-2 | TRBJ2-2 | CASSELNSGELFF | | | | | | | | 4 | | | | | | | | | | |
| TRAV5 | TRAJ13 | GGYQKVTF | TRBV3-1/TRBV3-2 | TRBJ2-2 | CASQPLNTGELFF | | | | | | | | 1 | | | | | | | | | | |
| TRAV12-1 | TRAJ43 | CVVNNYDDMRF | TRBV20-1/TRBV20/OR9-2 | TRBJ2-2 | CSAQIRNTGELFF | | | | | | | | | 1 | | | | | | | | | |
| TRAV-ND | TRAV-ND | TRAV-ND | TRBV10-2 | TRBJ2-2 | CASMVDENTGELFF | | | | | | | | | | 1 | | | | | | | | |
| TRAV-ND | TRAV-ND | TRAV-ND | TRBV2 | TRBJ2-2 | CASSDLDTGELFF | | | | | | | | | | 1 | | | | | | | | |
| TRAV-ND | TRAV-ND | TRAV-ND | TRBV7-9 | TRBJ1-1 | CASSPDIEAFF | | | | | | | | | | 1 | | | | | | | | |
| TRAV12-1 | TRAJ30 | CAVNRADKIIF | TRBV7-9 | TRBJ1-1 | CASSPDIEAFF | | | | | | | | | | 1 | | | | | | | | |
| TRAV12-1 | TRAJ30 | CAVNRDDKIIF | TRBV2 | TRBJ2-2 | CASQDRNTGELFF | | | | | | | | | | 1 | | | | | | | | |
| TRAV12-1 | TRAJ34 | CAGNQGDKLIF | TRBV7-9 | TRBJ1-1 | CASSGDVEAFF | | | | | | | | | | 1 | | | | | | | | |
| TRAV12-2 | TRAJ30 | CAVNRDDKIIF | TRBV20-1 | TRBJ2-2 | CSARDHQAQNTGELFF | | | | | | | | | | 3 | 1 | | | | | | | |
| TRAV13-2 | TRAJ31 | CAENARLMF | TRBV7-9 | TRBJ1-1 | CASSSDIEAFF | | | | | | | | | | 1 | | | | | | | | |
| TRAV8-4 | TRAJ53 | CAVPGGGGSNYKLTF | TRBV15 | TRBJ2-2 | CATWEANTGELFF | | | | | | | | | | 1 | | | | | | | | |
| TRAV-ND | TRAV-ND | TRAV-ND | TRBV2 | TRBJ2-2 | CASSDLNSGELFF | | | | | | | | | | | 1 | | | | | | | |
| TRAV-ND | TRAV-ND | TRAV-ND | TRBV7-9 | TRBJ1-1 | CASSTDIEAFF | | | | | | | | | | | 1 | | | | | | | |
| TRAV12-1 | TRAJ53 | CAVNDRDKLTF | TRBV7-9 | TRBJ1-1 | CASSTDIEAFF | | | | | | | | | | | 1 | | | | | | | |
| TRAV12-2 | TRAJ30 | CAVNRDDKIIF | TRBV20-1/TRBV20/OR9-2 | TRBJ2-2 | CSARDHQAQNTGELFF | | | | | | | | | | | 1 | | | | | | | |
| TRAV12-2 | TRAJ8 | CAVSGFQKLVF | TRBV4-1 | TRBJ1-1 | CASSQDGGGQLAFF | | | | | | | | | | | 1 | | | | | | | |
| TRAV-ND | TRAV-ND | TRAV-ND | TRBV14 | TRBJ2-2 | CASRDIGQANTGELFF | | | | | | | | | | | | 1 | | | | | | |
| TRAV-ND | TRAV-ND | TRAV-ND | TRBV19 | TRBJ2-2 | CAGQGLNTGELFF | | | | | | | | | | | | 1 | | | | | | |
| TRAV12-1 | TRAJ43 | CVVNGGNDMRF | TRBV2 | TRBJ2-2 | CASSDLNTGELFF | | | | | | | | | | | | 1 | | | | | | |
| TRAV19 | TRAJ26 | CALSEDNYGQNFVF | TRBV27 | TRBJ1-4 | CASSHGHWEKLFF | | | | | | | | | | | | 1 | | | | | | |
| TRAV-ND | TRAV-ND | TRAV-ND | TRBV7-8 | TRBJ2-2 | CASRDENTGELFF | | | | | | | | | | | | | 1 | | | | | |
| TRAV12-1 | TRAJ34 | CVVNEHDKLIF | TRBV7-9 | TRBJ1-1 | CASSGDIEAFF | | | | | | | | | | | | | 1 | | | | | |
| TRAV12-1 | TRAJ34 | CVVNGADKLIF | TRBV7-9 | TRBJ2-2 | CARGNANTGELFF | | | | | | | | | | | | | 1 | | | | | |
| TRAV12-2 | TRAJ34 | CAVGPDKLIF | TRBV-ND | TRBV-ND | TRBV-ND | | | | | | | | | | | | | 1 | | | | | |
| TRAV12-2 | TRAJ44 | CAVLTGTASKLTF | TRBV10-3 | TRBJ2-3 | CAISVGPTDTQYF | | | | | | | | | | | | | 1 | | | | | |
| TRAV26-2 | TRAJ40 | CILRGGNSGTYKYIF | TRBV19 | TRBJ2-2 | CASQTQNTGELFF | | | | | | | | | | | | | 1 | | | | | |
| TRAV-ND | TRAV-ND | TRAV-ND | TRBV7-9 | TRBJ2-7 | CASGLDSEQYF | | | | | | | | | | | | | | 1 | | | | |
| TRAV-ND | TRAV-ND | TRAV-ND | TRBV12-3/TRBV12-4 | TRBJ2-2 | CALLALNTGELFF | | | | | | | | | | | | | | | 1 | | | |
| TRAV-ND | TRAV-ND | TRAV-ND | TRBV30 | TRBJ2-2 | CAFADRNTGELFF | | | | | | | | | | | | | | | 1 | | | |
| TRAV-ND | TRAV-ND | TRAV-ND | TRBV7-8 | TRBJ2-2 | CASGHLNTGELFF | | | | | | | | | | | | | | | 1 | | | |
| TRAV9-2 | TRAJ22 | CALSAFRPGSARQLTF | TRBV24-1/TRBV24/OR9-2 | TRBJ2-2 | CATSDLNTGELFF | | | | | | | | | | | | | | | 4 | 1 | | |
| TRAV-ND | TRAV-ND | TRAV-ND | TRBV24-1/TRBV24/OR9-2 | TRBJ2-2 | CATSDLNTGELFF | | | | | | | | | | | | | | | | 1 | | |
| TRAV12-1 | TRAJ41 | CVVNMEDALNF | TRBV30 | TRBJ2-2 | CAFADRNTGELFF | | | | | | | | | | | | | | | | 1 | | |
| TRAV-ND | TRAV-ND | TRAV-ND | TRBV20-1/TRBV20/OR9-2 | TRBJ2-2 | CSARDELATNTGELFF | | | | | | | | | | | | | | | | | 2 | |
| TRAV-ND | TRAV-ND | TRAV-ND | TRBV20-1 | TRBJ2-2 | CSAPRDLNTGELFF | | | | | | | | | | | | | | | | | 1 | |
| TRAV-ND | TRAV-ND | TRAV-ND | TRBV20-1 | TRBJ2-2 | CSARDELATNTGELFF | | | | | | | | | | | | | | | | | 2 | |
| TRAV-ND | TRAV-ND | TRAV-ND | TRBV28 | TRBJ1-1 | CASSLKNSLEAFF | | | | | | | | | | | | | | | | | 1 | |
| TRAV-ND | TRAV-ND | TRAV-ND | TRBV5-4/TRBV5-8 | TRBJ2-2 | CASMADRNTGELFF | | | | | | | | | | | | | | | | | 1 | |
| TRAV-ND | TRAV-ND | TRAV-ND | TRBV7-8 | TRBJ2-2 | CARGVLNTGELFF | | | | | | | | | | | | | | | | | 1 | |
| TRAV-ND | TRAV-ND | TRAV-ND | TRBV7-9 | TRBJ2-7 | CASSPDIEQYF | | | | | | | | | | | | | | | | | 2 | |
| TRAV12-1 | TRAJ34 | CVVNENDKLIF | TRBV-ND | TRBV-ND | TRBV-ND | | | | | | | | | | | | | | | | | 1 | |
| TRAV12-1 | TRAJ34 | CVVNENDKLIF | TRBV7-9 | TRBJ1-2 | CASSPDIGYTF | | | | | | | | | | | | | | | | | 1 | |
| TRAV12-1 | TRAJ43 | CVVNNNNDMRF | TRBV6-5 | TRBJ2-2 | CAIQELNTGELFF | | | | | | | | | | | | | | | | | 1 | |
| TRAV12-2 | TRAJ30 | CAVNEDDKIIF | TRBV7-9 | TRBJ2-7 | CASSPDIKQYF | | | | | | | | | | | | | | | | | 1 | |
| TRAV12-2 | TRAJ45 | CAVNGWSGGGADGLTF | TRBV7-9 | TRBJ1-1 | CASSPDIEAFF | | | | | | | | | | | | | | | | | 1 | |
| TRAV13-2 | TRAJ26 | CAENISYGQNFVF | TRBV7-9 | TRBJ1-1 | CASSPDIEAFF | | | | | | | | | | | | | | | | | 1 | |
| TRAV19 | TRAJ52 | CALSQEAGGTSYGKLTF | TRBV13 | TRBJ1-2 | CASSFQGFTRGYTF | | | | | | | | | | | | | | | | | 1 | |
| TRAV35 | TRAJ35 | CAGQLSLVGRGNVLHC | TRBV2 | TRBJ2-2 | CASSELNTGELFF | | | | | | | | | | | | | | | | | 1 | |
| TRAV5 | TRAJ26 | CAEILDNYGQNFVF | TRBV3-1/TRBV3-2 | TRBJ2-7 | CASSQDAGYEQYF | | | | | | | | | | | | | | | | | 1 | |

| | | | | | | | | | | | | | | | | | | | | | | | | | |
|---|---|---|---|---|---|---|---|---|---|---|---|---|---|---|---|---|---|---|---|---|---|---|---|---|---|
| TRAV5 | TRAJ9 | CALDTGGFKTIF | TRBV27 | TRBJ2-2 | CASTEGNTGELFF | | | | | | | | | | | | | | | | | 1 | | | |
| TRAV-ND | TRAV-ND | TRAV-ND | TRBV19 | TRBJ1-5 | CASSIGRNYDNQPQHF | | | | | | | | | | | | | | | | | | 1 | | |

| A24/$S_{1208}$ | | | | | Donor | 7 | 7 | 7 | 7 | 15 | 15 | 17 | 17 | 17 | 22 | 22 | 23 | 23 | 27 | 28 | 39 | 39 | 41 | 51 | 51 |
|---|---|---|---|---|---|---|---|---|---|---|---|---|---|---|---|---|---|---|---|---|---|---|---|---|---|
| | | | | | Long COVID | Y | Y | Y | Y | N | N | Y | Y | N | Y | Y | Y | Y | Y | Y | Y | Y | Y | Y | Y |
| | | | | | Timepoint (months) | ac | 18 | 24 | 6 | 3 | 18 | 12 | 24 | 12 | 12 | 24 | 3 | 12 | 12 | 12 | 3 | 12 | 3 | 12 | 24 |
| TRAV | TRAJ | CDR3α | TRBV | TRBJ | CDR3β | | | | | | | | | | | | | | | | | | | | |
| TRAV12-2 | TRAJ47 | CAVDGNKLVF | TRBV2 | TRBJ2-7 | CASSQGSYEQYF | 1 | | | | | | | | | | | | | | | | | | | |
| TRAV17 | TRAJ52 | CATDDFLSGTSYGKLTF | TRBV20-1 | TRBJ2-7 | CSARDQEKAYEQYF | 1 | 5 | 1 | | | | | | | | | | | | | | | | | |
| TRAV8-6 | TRAJ21 | CAVSGGDNFNKFYF | TRBV20-1 | TRBJ1-2 | CSARDIGQAYSYTF | 1 | | | | | | | | | | | | | | | | | | | |
| TRAV-ND | TRAV-ND | TRAV-ND | TRBV20-1 | TRBJ1-2 | CSARDIGQAYSYTF | | | | 1 | | | | | | | | | | | | | | | | |
| TRAV-ND | TRAV-ND | TRAV-ND | TRBV27 | TRBJ1-5 | CASSLWPVGNQPQHF | | | | 1 | | | | | | | | | | | | | | | | |
| TRAV-ND | TRAV-ND | TRAV-ND | TRBV29-1 | TRBJ2-3 | CSVDLDWPRDTQYF | | | | 1 | | | | | | | | | | | | | | | | |
| TRAV-ND | TRAV-ND | TRAV-ND | TRBV29-1 | TRBJ2-7 | CSVLGDSAQYEQYF | | | | 1 | | | | | | | | | | | | | | | | |
| TRAV-ND | TRAV-ND | TRAV-ND | TRBV2 | TRBJ2-1 | CASNQRESNEQFF | | | | 1 | | | | | | | | | | | | | | | | |
| TRAV-ND | TRAV-ND | TRAV-ND | TRBV7-9 | TRBJ1-6 | CASSLVGSGSYNSPLHF | | | | 1 | | | | | | | | | | | | | | | | |
| TRAV17 | TRAJ48 | CATENDFGNEKLTF | TRBV15 | TRBJ1-4 | CATSNLGGEKLFF | | | | 1 | | | | | | | | | | | | | | | | |
| TRAV24 | TRAJ13 | CASSYQKVTF | TRBV5-6 | TRBJ1-1 | CASSLAGVNTEAFF | | | | 1 | | | | | | | | | | | | | | | | |
| TRAV27 | TRAJ30 | CAGGNRDDKIIF | TRBV28 | TRBJ1-1 | CASSLSETEAFF | | | | 1 | | | | | | | | | | | | | | | | |
| TRAV35 | TRAJ37 | CAGNTGKLIF | TRBV19 | TRBJ2-1 | CASSIGRHNEQFF | | | | 1 | | | | | | | | | | | | | | | | |
| TRAV35 | TRAJ44 | CAGRLSGTASKLTF | TRBV27 | TRBJ2-7 | CASSLSSSGSYEQYF | | | | 1 | | | | | | | | | | | | | | | | |
| TRAV-ND | TRAV-ND | TRAV-ND | TRBV5-6 | TRBJ1-1 | CASSPQMSEAFF | | 1 | 2 | | | | | | | | | | | | | | | | | |
| TRAV17 | TRAJ52 | CATDDFLSGTSYGKLTF | TRBV20-1/TRBV20/OR9-2 | TRBJ2-7 | CSARDQEKAYEQYF | | 2 | | | | | | | | | | | | | | | | | | |
| TRAV19 | TRAJ16 | CALSEGSDGGQKLLF | TRBV20-1 | TRBJ2-7 | CSAGPLGRNYEQYF | | 1 | | | | | | | | | | | | | | | | | | |
| TRAV19 | TRAJ22 | CALSGLPPARQLTF | TRBV20-1/TRBV20/OR9-2 | TRBJ2-7 | CSAETSGSNYEQYF | | 1 | | | | | | | | | | | | | | | | | | |
| TRAV19 | TRAJ22 | CALSGLPPARQLTF | TRBV20-1 | TRBJ2-7 | CSAETSGSNYEQYF | | 1 | | | | | | | | | | | | | | | | | | |
| TRAV6 | TRAJ10 | CALDGLTGGGNKLTF | TRBV2 | TRBJ2-3 | CASSGRDSLTDTQYF | | 1 | | | | | | | | | | | | | | | | | | |
| TRAV-ND | TRAV-ND | TRAV-ND | TRBV7-6 | TRBJ1-4 | CASSPTQVHGEKLFF | | | 1 | | | | | | | | | | | | | | | | | |
| TRAV24 | TRAJ20 | CAPLPYNDYKLSF | TRBV7-6 | TRBJ1-4 | CASSPTQVHGEKLFF | | | 1 | | | | | | | | | | | | | | | | | |
| TRAV29/DV5 | TRAJ39 | CAASGVGNAGNMLTF | TRBV7-9 | TRBJ2-5 | CASDPGLAGYQETQYF | | | 1 | | | | | | | | | | | | | | | | | |
| TRAV-ND | TRAV-ND | TRAV-ND | TRBV5-6 | TRBJ2-3/TRBJ2-4 | CASSPDGTGGNIQYF | | | | | 1 | | | | | | | | | | | | | | | |
| TRAV21 | TRAJ37 | CAVWDTGKLIF | TRBV15 | TRBJ2-1 | CATSSDQGSEQFF | | | | | 1 | | | | | | | | | | | | | | | |
| TRAV24 | TRAJ13 | CARNSGGYQKVTF | TRBV5-6 | TRBJ2-1 | CASSAQVNEQFF | | | | | 1 | | | | | | | | | | | | | | | |
| TRAV-ND | TRAV-ND | TRAV-ND | TRBV10-2 | TRBJ1-4 | CASQHQPNEKLFF | | | | | | 3 | | | | | | | | | | | | | | |
| TRAV-ND | TRAV-ND | TRAV-ND | TRBV2 | TRBJ2-2 | CASSAGADTGELFF | | | | | | 3 | | | | | | | | | | | | | | |
| TRAV-ND | TRAV-ND | TRAV-ND | TRBV5-6 | TRBJ2-1 | CASSAQVNEQFF | | | | | | 1 | | | | | | | | | | | | | | |
| TRAV1-2 | TRAJ20 | CACCYNDYKLSF | TRBV12-3/TRBV12-4 | TRBJ2-7 | CASSFGGAAYEQYF | | | | | | 1 | | | | | | | | | | | | | | |
| TRAV21 | TRAJ13 | CAVRLTQGGYQKVPF | TRBV5-6 | TRBJ2-2 | CASSQGAAGELFF | | | | | | 1 | | | | | | | | | | | | | | |
| TRAV21 | TRAJ13 | CAVRLTQGGYQKVTF | TRBV5-6 | TRBJ2-2 | CASSQGAAGELFF | | | | | | 2 | | | | | | | | | | | | | | |
| TRAV21 | TRAJ43 | CAVPRYNNNDMRF | TRBV2 | TRBJ2-2 | CASSAGADTGELFF | | | | | | 2 | | | | | | | | | | | | | | |
| TRAV21 | TRAJ9 | CAGVGTGGFKTIF | TRBV5-6 | TRBJ2-1 | CASSLLSNEQFF | | | | | | 3 | | | | | | | | | | | | | | |
| TRAV23/DV6 | TRAJ21 | CAAGLSYKFNKFYF | TRBV5-6 | TRBJ2-3 | CASSSSGGYTDTQYF | | | | | | 1 | | | | | | | | | | | | | | |
| TRAV23/DV6 | TRAJ21 | CAAGLSYNFNKFYF | TRBV5-6 | TRBJ2-3 | CASSSSGGYTDTQYF | | | | | | 2 | | | | | | | | | | | | | | |
| TRAV26-1 | TRAJ17 | GNKLTF | TRBV5-6 | TRBJ2-1 | CASSLGGGGRYNEQFF | | | | | | 1 | | | | | | | | | | | | | | |
| TRAV29/DV5 | TRAJ31 | CAASDARLMF | TRBV20-1/TRBV20/OR9-2 | TRBJ2-7 | CSARDIVAGGHYEQYF | | | | | | 1 | | | | | | | | | | | | | | |
| TRAV30 | TRAJ20 | CGTSGLNDYKLSF | TRBV10-2 | TRBJ1-4 | CASQHQPNEKLFF | | | | | | 2 | | | | | | | | | | | | | | |
| TRAV38-2/DV8 | TRAJ48 | CAYRSNYPADGNEKLTF | TRBV20-1 | TRBJ2-1 | CSARDIAAGRYNEQFF | | | | | | 1 | | | | | | | | | | | | | | |
| TRAV-ND | TRAV-ND | TRAV-ND | TRBV4-3 | TRBJ2-2 | CASSSDRAVTGELFF | | | | | | | 1 | | | | | | | | | | | | | |
| TRAV1-2 | TRAJ33 | CAVSDSNYQLIW | TRBV29-1 | TRBJ2-2 | CSVTLPGQDTGELFF | | | | | | | | 1 | | | | | | | | | | | | |
| TRAV24 | TRAJ53 | CAFPRGGSNYKLTF | TRBV20-1 | TRBJ2-7 | CSARDTAVGGHYEQYF | | | | | | | | 1 | | | | | | | | | | | | |
| TRAV41 | TRAJ34 | CAVRSYNTDKLIF | TRBV-ND | TRBV-ND | TRBV-ND | | | | | | | | 1 | | | | | | | | | | | | |
| TRAV-ND | TRAV-ND | TRAV-ND | TRBV15 | TRBJ2-2 | CATSRVAGNTGELFF | | | | | | | | | 1 | | | | | | | | | | | |
| TRAV-ND | TRAV-ND | TRAV-ND | TRBV19 | TRBJ2-3 | CASRAPGLAGQIEDTQYF | | | | | | | | | | 1 | | | | | | | | | | |
| TRAV-ND | TRAV-ND | TRAV-ND | TRBV25-1 | TRBJ2-3 | CASSEGTSGTDTQYF | | | | | | | | | | 1 | | | | | | | | | | |
| TRAV-ND | TRAV-ND | TRAV-ND | TRBV5-6 | TRBJ2-5 | CASSQSGGETQYF | | | | | | | | | | 1 | | | | | | | | | | |
| TRAV14/DV4 | TRAJ28 | CAMRDLSGAGSYQLTF | TRBV19 | TRBJ2-3 | CASRAPGLAGQIEDTQYF | | | | | | | | | | 1 | | | | | | | | | | |
| TRAV8-2 | TRAJ45 | CVVRPYSGGGADGLTF | TRBV5-6 | TRBJ2-5 | CASSQSGGETQYF | | | | | | | | | | 1 | | | | | | | | | | |
| TRAV8-3 | TRAJ54 | CAANLTFFIIQGAQKLVF | TRBV5-6 | TRBJ2-5 | CASSLDGTGSKTQYF | | | | | | | | | | 1 | | | | | | | | | | |
| TRAV8-6 | TRAJ4 | CAVTGEGGYNKLIF | TRBV20-1/TRBV20/OR9-2 | TRBJ2-7 | CSARDLGQAYEQYF | | | | | | | | | | 1 | | | | | | | | | | |
| TRAV-ND | TRAV-ND | TRAV-ND | TRBV2 | TRBJ2-3 | CARTGLAGDTQYF | | | | | | | | | | | 1 | | | | | | | | | |
| TRAV29/DV5 | TRAJ44 | CAASVTGTASKLTF | TRBV13 | TRBJ2-1 | CASSATGLVPTYNEQFF | | | | | | | | | | | 1 | | | | | | | | | |
| TRAV-ND | TRAV-ND | TRAV-ND | TRBV10-3 | TRBJ1-3 | CAISDPLTGDFGNTIYF | | | | | | | | | | | | 1 | | | | | | | | |
| TRAV-ND | TRAV-ND | TRAV-ND | TRBV20-1/TRBV20/OR9-2 | TRBJ2-1 | CSARDQGLAGLEQFF | | | | | | | | | | | | | 1 | | | | | | | |
| TRAV-ND | TRAV-ND | TRAV-ND | TRBV5-6 | TRBJ2-2 | CASILGTPRTGELFF | | | | | | | | | | | | | 2 | | | | | | | |
| TRAV-ND | TRAV-ND | TRAV-ND | TRBV5-6 | TRBJ2-6 | CASSLGTGYSGANVLTF | | | | | | | | | | | | | 1 | | | | | | | |
| TRAV12-1 | TRAJ16 | CAVTPFSDGQKLLF | TRBV20-1/TRBV20/OR9-2 | TRBJ2-7 | CSARDGGLAGPEQYF | | | | | | | | | | | | | 1 | | | | | | | |
| TRAV16 | TRAJ32 | CAPGPRRGGATNKLIF | TRBV20-1 | TRBJ2-7 | CSAQPTGRNYEQYF | | | | | | | | | | | | | 1 | | | | | | | |
| TRAV24 | TRAJ53 | CAHSGGSNYKLTF | TRBV5-6 | TRBJ1-2 | CASSLGTPAYGYTF | | | | | | | | | | | | | 1 | | | | | | | |
| TRAV8-2/TRAV8-4 | TRAJ16 | CAVSDIGSKLLF | TRBV5-6 | TRBJ2-1 | CASSLAGGGEQFF | | | | | | | | | | | | | 1 | | | | | | | |
| TRAV8-4 | TRAJ16 | CAVSDIGSKLLF | TRBV5-6 | TRBJ2-1 | CASSLAGGGEQFF | | | | | | | | | | | | | 2 | | | | | | | |
| TRAV1-2 | TRAJ40 | CAVRSTTSGTYKYIF | TRBV10-1 | TRBJ2-7 | CASSQQPTGAEQYF | | | | | | | | | | | | | | 1 | | | | | | |
| TRAV13-1 | TRAJ5 | CALDLRGRPSFDTGRRALTF | TRBV27 | TRBJ1-1 | CASSAGVLNTEAFF | | | | | | | | | | | | | | 1 | | | | | | |
| TRAV26-1 | TRAJ22 | CIVDPWGQLTF | TRBV2 | TRBJ2-7 | CASSEGLAGPYEQYF | | | | | | | | | | | | | | 1 | | | | | | |
| TRAV4 | TRAJ45 | CLVTGGGADGLTF | TRBV-ND | TRBV-ND | TRBV-ND | | | | | | | | | | | | | | 1 | | | | | | |
| TRAV-ND | TRAV-ND | TRAV-ND | TRBV7-9 | TRBJ1-6 | CASSLAENNSPLHF | | | | | | | | | | | | | | | 1 | | | | | |
| TRAV-ND | TRAV-ND | TRAV-ND | TRBV20-1/TRBV20/OR9-2 | TRBJ2-7 | CSARDLGTHEQYF | | | | | | | | | | | | | | | | 1 | | | | |
| TRAV-ND | TRAV-ND | TRAV-ND | TRBV20-1/TRBV20/OR9-2 | TRBJ2-7 | CSARDVGQAYEQYF | | | | | | | | | | | | | | | | 1 | | | | |
| TRAV19 | TRAJ52 | CALSDLFNAGGTSYGKLTF | TRBV20-1/TRBV20/OR9-2 | TRBJ2-7 | CSARDGRGAGPGEQYF | | | | | | | | | | | | | | | | 1 | | | | |
| TRAV21 | TRAJ49 | CAVNPNTGNQFYF | TRBV11-3 | TRBJ2-5 | CASSLGLAGIFQETQYF | | | | | | | | | | | | | | | | 3 | 1 | | | |
| TRAV-ND | TRAV-ND | TRAV-ND | TRBV5-5 | TRBJ1-4 | CASSPWTGTNEKLFF | | | | | | | | | | | | | | | | | 1 | | | |
| TRAV19 | TRAJ34 | CALRLYNTDKLIF | TRBV20-1 | TRBJ2-7 | CSARDLGRYEQYF | | | | | | | | | | | | | | | | | 1 | | | |
| TRAV-ND | TRAV-ND | TRAV-ND | TRBV20-1/TRBV20/OR9-2 | TRBJ2-7 | CSATPTSGGRHYEQYF | | | | | | | | | | | | | | | | | | 1 | | |
| TRAV-ND | TRAV-ND | TRAV-ND | TRBV4-1 | TRBJ2-7 | CASSQEVWTNYEQYF | | | | | | | | | | | | | | | | | | | 1 | |
| TRAV8-1 | TRAJ44 | CAVEEDGTASKLTF | TRBV29-1 | TRBJ1-2 | CSVDSGQNSNYGYTF | | | | | | | | | | | | | | | | | | | | 1 |

| A3/$S_{378}$ | | | | | Donor | 6 | 6 | 6 | 11 | 11 |
|---|---|---|---|---|---|---|---|---|---|---|
| | | | | | Long COVID | Y | Y | Y | N | N |
| | | | | | Timepoint (months) | ac | 6 | 24 | ac | 12 |
| TRAV | TRAJ | CDR3α | TRBV | TRBJ | CDR3β | | | | | |
| TRAV13-1 | TRAJ11 | CAASIRSGYSTLTF | TRBV4-1 | TRBJ2-5 | CASSQEGKQETQYF | 1 | | | | |
| TRAV4 | TRAJ22 | CLVGDNIAGSARQLTF | TRBV28 | TRBJ2-7 | CASSLPLPTGTYEQYF | 1 | | | | |
| TRAV6 | TRAJ23 | CALDGNQGGKLIF | TRBV10-3 | TRBJ2-5 | CAISESVSEETQYF | 1 | | | | |
| TRAV9-2 | TRAJ29 | CAHGGNTPLVF | TRBV27 | TRBJ2-7 | CASSLSGTYEQYF | 1 | | | | |
| TRAV-ND | TRAV-ND | TRAV-ND | TRBV24-1/TRBV24/OR9-2 | TRBJ2-1 | CATSVWDRGHNEQFF | | 1 | | | |
| TRAV-ND | TRAV-ND | TRAV-ND | TRBV9 | TRBJ1-2 | CASSVGGFDYGYTF | | 1 | | | |
| TRAV1-2 | TRAJ33 | CAVTDSNYQLIW | TRBV-ND | TRBV-ND | TRBV-ND | | 1 | | | |
| TRAV14/DV4 | TRAJ23 | CAMREVYNQGGKLIF | TRBV4-1 | TRBJ1-5 | CASSQDAGQGTQPQHF | | 1 | | | |
| TRAV21 | TRAJ27 | CAVDTNAGKSTF | TRBV12-3/TRBV12-4 | TRBJ2-2 | CASSLLARPNTGELFF | | 1 | | | |
| TRAV26-1 | TRAJ39 | CIVRDNNAGNMLTF | TRBV9 | TRBJ2-1 | CASSVDGRGGDEQFF | | 1 | | | |
| TRAV5 | TRAJ44 | CAESTGTASKLTF | TRBV9 | TRBJ1-2 | CASSTPTGIYGYTF | | 1 | | | |
| TRAV6 | TRAJ33 | CALSSNYQLIW | TRBV28 | TRBJ2-7 | CASSLRGGSSYEQYF | | 1 | | | |
| TRAV-ND | TRAV-ND | TRAV-ND | TRBV19 | TRBJ2-5 | CASSITATRETQYF | | | 1 | | |
| TRAV-ND | TRAV-ND | TRAV-ND | TRBV5-5 | TRBJ1-2 | CASSLGAGAGYGYTF | | | 1 | | |
| TRAV-ND | TRAV-ND | TRAV-ND | TRBV9 | TRBJ1-1 | CASTPWGGNTEAFF | | | 5 | | |
| TRAV1-2 | TRAJ23 | CAVRYNQGGKLIF | TRBV-ND | TRBV-ND | TRBV-ND | | | 1 | | |
| TRAV10 | TRAJ39 | CVVRTNNAGNMLTF | TRBV4-2 | TRBJ1-5 | CASSQEIGLWDQPQHF | | | 1 | | |

| | | | | | | | | | | | | | | | |
|---|---|---|---|---|---|---|---|---|---|---|---|---|---|---|---|
| TRAV10 | TRAJ39 | CVVSNNNAGNMLTF | TRBV4-2 | TRBJ1-6 | CASSQEVGDRHSPLHF | | | 1 | | | | | | | |
| TRAV10 | TRAJ6 | CVVSERGSYIPTF | TRBV25-1 | TRBJ1-2 | CASSEKNYGYTF | | | 2 | | | | | | | |
| TRAV12-2 | TRAJ52 | CAVNGGTSYGKLTF | TRBV9 | TRBJ2-2 | CASSVKGLGENTGELFF | | | 1 | | | | | | | |
| TRAV13-1 | TRAJ15 | CAALPSDQAGTALIF | TRBV27 | TRBJ1-1 | CASGVTRSGTEAFF | | | 2 | | | | | | | |
| TRAV13-2 | TRAJ15 | CAENMSNQAGTALIF | TRBV-ND | TRBV-ND | TRBV-ND | | | 2 | | | | | | | |
| TRAV14/DV4 | TRAJ21 | CAMILGNFNKFYF | TRBV12-3/TRBV12-4 | TRBJ2-1 | CASTSIHSGGRGNEQFF | | | 1 | | | | | | | |
| TRAV14/DV4 | TRAJ29 | CAMREGESGNTPLVF | TRBV4-2 | TRBJ1-5 | CASSQELRVRNQPQHF | | | 1 | | | | | | | |
| TRAV14/DV4 | TRAJ30 | CAMRGPNRDDKIIF | TRBV4-2 | TRBJ1-5 | CASSQEQGVRNQPQHF | | | 2 | | | | | | | |
| TRAV16 | TRAJ54 | PGGAQKLVF | TRBV-ND | TRBV-ND | TRBV-ND | | | 2 | | | | | | | |
| TRAV26-1 | TRAJ15 | CIVSSNQAGTALIF | TRBV9 | TRBJ2-3 | CASSEYGGSDTQYF | | | 1 | | | | | | | |
| TRAV26-1 | TRAJ39 | CIVRDNNAGNMLTF | TRBV9 | TRBJ1-1 | CASTPWGGNTEAFF | | | 2 | | | | | | | |
| TRAV26-2 | TRAJ50 | CILRDKGTSYDKVIF | TRBV4-2 | TRBJ2-2 | CASSQTIGSARNTGELFF | | | 2 | | | | | | | |
| TRAV30 | TRAJ37 | CGTEILSGNTGKLIF | TRBV30 | TRBJ1-1 | CAWRWVKNTEAFF | | | 1 | | | | | | | |
| TRAV-ND | TRAV-ND | TRAV-ND | TRBV19 | TRBJ2-1 | CASSIKLAGGPWNEQFF | | | | 1 | | | | | | |
| TRAV-ND | TRAV-ND | TRAV-ND | TRBV19 | TRBJ2-7 | CASSFKQGAGEQYF | | | | 1 | | | | | | |
| TRAV-ND | TRAV-ND | TRAV-ND | TRBV29-1 | TRBJ2-2 | CSVLPSGRFTGELFF | | | | 3 | | | | | | |
| TRAV-ND | TRAV-ND | TRAV-ND | TRBV4-1 | TRBJ2-7 | CASSLPGGWEEQYF | | | | 1 | | | | | | |
| TRAV-ND | TRAV-ND | TRAV-ND | TRBV4-2 | TRBJ2-2 | CASSQEWRELGTGELFF | | | | 1 | | | | | | |
| TRAV-ND | TRAV-ND | TRAV-ND | TRBV4-2 | TRBJ2-5 | CASSQERGSILETQYF | | | | 1 | | | | | | |
| TRAV-ND | TRAV-ND | TRAV-ND | TRBV9 | TRBJ2-7 | CASSVYGRGEQYF | | | | 1 | | | | | | |
| TRAV1-2 | TRAJ15 | CAVSQQAGTALIF | TRBV24-1/TRBV24/OR9-2 | TRBJ2-1 | CATSARTAYNEQFF | | | | 1 | | | | | | |
| TRAV12-2 | TRAJ39 | CAVNSNNAGNMLTF | TRBV19 | TRBJ1-1 | CASSIKQGTEAFF | | | | 1 | | | | | | |
| TRAV12-2 | TRAJ43 | CAVRDNNDMRF | TRBV4-2 | TRBJ2-7 | CASSQESRTYEQYF | | | | 1 | | | | | | |
| TRAV13-2 | TRAJ30 | CAGQYDKIIF | TRBV9 | TRBJ2-2 | CASSAWGSGELFF | | | | 1 | | | | | | |
| TRAV19 | TRAJ37 | CALSVPLHGSSNTGKLIF | TRBV4-2 | TRBJ2-5 | CASSQERGSILETQYF | | | | 1 | | | | | | |
| TRAV20 | TRAJ24 | CAVQESWGKLQF | TRBV28 | TRBJ2-3 | CASSLSRTDTQYF | | | | 1 | | | | | | |
| TRAV25 | TRAJ45 | CAGPSGGGADGLTF | TRBV12-3/TRBV12-4 | TRBJ1-5 | CASSLWYSRGNQPQHF | | | | 1 | | | | | | |
| TRAV26-1 | TRAJ39 | CIVMDNNAGNMLTF | TRBV19 | TRBJ1-1 | CASSIKQGTEAFF | | | | 1 | | | | | | |
| TRAV26-1 | TRAJ39 | CIVQNNNAGNMLTF | TRBV9 | TRBJ2-7 | CASSVYGRGEQYF | | | | 1 | | | | | | |
| TRAV26-1 | TRAJ45 | CIVRVADSGGGADGLTF | TRBV19 | TRBJ1-5 | CASTWAWRGRQPQHF | | | | 1 | | | | | | |
| TRAV26-2 | TRAJ39 | CILSDNNAGNMLTF | TRBV19 | TRBJ1-1 | CASSLKQGARGGAFF | | | | 1 | | | | | | |
| TRAV38-1 | TRAJ30 | CAFTVNRDDKIIF | TRBV7-9 | TRBJ2-7 | CASSFDQGAPYEQYF | | | | 1 | | | | | | |
| TRAV8-3 | TRAJ39 | CAVGTNNAGNMLTF | TRBV4-2 | TRBJ2-2 | CASSQEWRELGTGELFF | | | | 1 | | | | | | |
| TRAV8-6 | TRAJ43 | CAVSASNNDMRF | TRBV6-1 | TRBJ2-5 | CASGREPRDWGETQYF | | | | 1 | | | | | | |
| TRAV-ND | TRAV-ND | TRAV-ND | TRBV4-2 | TRBJ2-7 | CASSQEQGLVHYEQYF | | | | | 2 | | | | | |
| TRAV1-2 | TRAJ33 | CAVLDSNYQLIW | TRBV20-1 | TRBJ2-1 | CSVGTSGGPPNEQFF | | | | | 1 | | | | | |
| TRAV17 | TRAJ30 | CATHAGDDKIIF | TRBV28 | TRBJ1-5 | CASSLRGNQPQHF | | | | | 1 | | | | | |
| TRAV19 | TRAJ26 | CALSERDNYGQNFVF | TRBV-ND | TRBV-ND | TRBV-ND | | | | | 1 | | | | | |
| TRAV36/DV7 | TRAJ23 | CANHEGGKLIF | TRBV29-1 | TRBJ2-1 | CSVDGLHGRSYNEQFF | | | | | 1 | | | | | |
| TRAV5 | TRAJ32 | CAESIREYGGATNKLIF | TRBV19 | TRBJ1-3 | CASRLGGQGPIGGNTIYF | | | | | 1 | | | | | |

| B35/$S_{321}$ | | | | | Donor | 13 | 13 | 13 | 3 | 3 | 3 | 3 | 5 | 5 | 9 |
|---|---|---|---|---|---|---|---|---|---|---|---|---|---|---|---|
| | | | | | Long COVID | Y | Y | Y | Y | Y | Y | Y | Y | Y | Y |
| | | | | | Timepoint | ac | 12 | 18 | 3 | 12 | 18 | 24 | 18 | 24 | 24 |
| TRAV | TRAJ | CDR3α | TRBV | TRBJ | CDR3β | | | | | | | | | | |
| TRAV-ND | TRAV-ND | TRAV-ND | TRBV4-3 | TRBJ1-1 | CASSQEPEPNTEAFF | | | | 1 | | | | | | |
| TRAV14/DV4 | TRAJ52 | CAMRALQNAGGTSYGKLTF | TRBV5-5 | TRBJ2-1 | CASAWAAASKGNEQFF | | | | | 1 | | | | | |
| TRAV-ND | TRAV-ND | TRAV-ND | TRBV4-3 | TRBJ2-1 | CASSQDRFLAEAPNEQFF | | | | | | 1 | | | | |
| TRAV10 | TRAJ8 | CVVVTGFQKLVF | TRBV12-3/TRBV12-4 | TRBJ2-5 | CASSWAGGPSEGETQYF | | | | | | 2 | | | | |
| TRAV12-2 | TRAJ7 | CAVYGNNRLAF | TRBV-ND | TRBV-ND | TRBV-ND | | | | | | 1 | | | | |
| TRAV13-2 | TRAJ26 | CAESTGQNFVF | TRBV-ND | TRBV-ND | TRBV-ND | | | | | | 1 | | | | |
| TRAV22 | TRAJ26 | CAAHFYGQNFVF | TRBV5-6 | TRBJ2-1 | CASSSTESGGPLDEQFF | | | | | | 1 | | | | |
| TRAV26-1 | TRAJ22 | CIVRSFSGSARQLTF | TRBV7-9 | TRBJ2-3 | CASSSDTDTQYF | | | | | | 1 | | | | |
| TRAV5 | TRAJ28 | SAESTGAGSYQLTF | TRBV25-1 | TRBJ1-1 | CASSEGSTEAFF | | | | | | 1 | | | | |
| TRAV5 | TRAJ34 | CAEYNTDKLIF | TRBV24-1/TRBV24/OR9-2 | TRBJ1-1 | CATSEGSTEAFF | | | | | | 11 | 5 | | | |
| TRAV8-3 | TRAJ22 | CAVETRRQLTF | TRBV28 | TRBJ2-1 | CASSESSGSSGDEQFF | | | | | | 1 | | | | |
| TRAV-ND | TRAV-ND | TRAV-ND | TRBV20-1 | TRBJ2-7 | CSALDAGDTTLYEQYF | | | | | | | 3 | | | |
| TRAV-ND | TRAV-ND | TRAV-ND | TRBV25-1 | TRBJ1-1 | CASSEGSTEAFF | | | | | | | 1 | | | |
| TRAV-ND | TRAV-ND | TRAV-ND | TRBV7-9 | TRBJ2-3 | CASSSDTDTQYF | | | | | | | 1 | | | |
| TRAV5 | TRAJ28 | CAESTGAGSYQLTF | TRBV25-1 | TRBJ1-1 | CASSEGSTEAFF | | | | | | | 1 | | | |
| TRAV5 | TRAJ28 | CAESTGAGSYQLTF | TRBV25-1 | TRBJ1-1 | CASSEGSTEAFF | | | | | | | 1 | | | |
| TRAV5 | TRAJ34 | CAEYNTDKLIF | TRBV24-1/TRBV24/OR9-2 | TRBJ1-1 | CATSEGSTEAFF | | | | | | | 4 | | | |
| TRAV-ND | TRAV-ND | TRAV-ND | TRBV24-1/TRBV24/OR9-2 | TRBJ1-1 | CATSDGSTEAFF | | | | | | | | 1 | | |
| TRAV-ND | TRAV-ND | TRAV-ND | TRBV7-2 | TRBJ1-1 | CASSLEVYMSTEAFF | | | | | | | | 4 | | |
| TRAV10 | TRAJ45 | CVVSANWGGGADGLTF | TRBV2 | TRBJ2-4 | CASSDEGVRLTSGKNIQYF | | | | | | | | 1 | | |
| TRAV12-3 | TRAJ40 | CAMSAGGTYKYIF | TRBV-ND | TRBV-ND | TRBV-ND | | | | | | | | 2 | 1 | |
| TRAV13-1 | TRAJ52 | CAARKVDAGGTSYGKLTF | TRBV24-1/TRBV24/OR9-2 | TRBJ2-7 | CATSEGSTYEQYF | | | | | | | | 1 | | |
| TRAV22 | TRAJ15 | CAPDQAGTALIF | TRBV20-1/TRBV20/OR9-2 | TRBJ2-1 | CSARDEAGGPLAEQFF | | | | | | | | 1 | | |
| TRAV22 | TRAJ15 | CAPYQAGTALIF | TRBV20-1/TRBV20/OR9-2 | TRBJ2-1 | CSARDEAGGPLAEQFF | | | | | | | | 3 | 3 | |
| TRAV22 | TRAJ15 | CAPYQAGTALIF | TRBV20-1 | TRBJ2-1 | CSARDEAGGPLAEQFF | | | | | | | | 1 | 3 | |
| TRAV22 | TRAJ39 | CAVPVYTSAANKFTF | TRBV7-2 | TRBJ1-1 | CASSLEVYMSTEAFF | | | | | | | | 1 | | |
| TRAV22 | TRAJ40 | CAVPLFTSGTYKYIF | TRBV7-2 | TRBJ1-1 | CASSLEVYMSTEAFF | | | | | | | | 1 | 2 | |
| TRAV22 | TRAJ40 | CAVPLFTSGTYKYIF | TRBV7-2 | TRBJ1-1 | CASSLEVYMSTEAFF | | | | | | | | 7 | 2 | |
| TRAV-ND | TRAV-ND | TRAV-ND | TRBV7-2 | TRBJ1-1 | CASSLEVYMSTEAFF | | | | | | | | | 1 | |
| TRAV19 | TRAJ24 | CALSGSSWTDSWGKLQF | TRBV7-2 | TRBJ2-3 | CASSLGTLAGDTQYF | | | | | | | | | 1 | |
| TRAV22 | TRAJ15 | CAPYQAGTALIF | TRBV-ND | TRBV-ND | TRBV-ND | | | | | | | | | 1 | |
| TRAV5 | TRAJ34 | CAERNTDKLIF | TRBV-ND | TRBV-ND | TRBV-ND | | | | | | | | | 1 | |
| TRAV-ND | TRAV-ND | TRAV-ND | TRBV29-1 | TRBJ1-5 | CSVGEEWGSNQPQHF | | | | | | | | | | 1 |
| TRAV-ND | TRAV-ND | TRAV-ND | TRBV30 | TRBJ1-1 | CAWSVDGNTEAFF | | | | | | | | | | 1 |
| TRAV12-2 | TRAJ33 | CAVNMDSNYQLIW | TRBV19 | TRBJ2-7 | CASSTLGIAYEQYF | | | | | | | | | | 4 |
| TRAV26-2 | TRAJ41 | CILRDVHSGYALNF | TRBV30 | TRBJ1-1 | CAWSVDGNTEAFF | | | | | | | | | | 6 |
| TRAV26-2 | TRAJ41 | CILRDVHSGYALNF | TRBV30 | TRBJ1-1 | CAWSVDGNTEAFF | | | | | | | | | | 3 |
| TRAV12-2 | TRAJ53 | CAVNEGSNYKLTF | TRBV19 | TRBJ2-7 | CASSSLTASYEQYF | 1 | | | | | | | | | |
| TRAV-ND | TRAV-ND | TRAV-ND | TRBV20-1/TRBV20/OR9-2 | TRBJ1-2 | CSAIDPGQGHEGGYTF | | 15 | 14 | | | | | | | |
| TRAV-ND | TRAV-ND | TRAV-ND | TRBV20-1 | TRBJ1-2 | CSAIDPGQGHEGGYTF | | 12 | 11 | | | | | | | |
| TRAV1-2 | TRAJ26 | CAGGRNYGQNFVF | TRBV20-1/TRBV20/OR9-2 | TRBJ1-2 | CSAIDPGQGHEGGYTF | | 1 | 3 | | | | | | | |
| TRAV1-2 | TRAJ26 | CAGGRNYGQNFVF | TRBV20-1 | TRBJ1-2 | CSAIDPGQGHEGGYTF | | 3 | | | | | | | | |
| TRAV1-2 | TRAJ26 | CAGGRNYGQNFVF | TRBV-ND | TRBV-ND | TRBV-ND | | | 1 | | | | | | | |

| B7/$N_{105}$ | | | | | Donor | 15 | 15 | 15 | 15 | 6 | 6 | 6 | 6 | 11 | 11 |
|---|---|---|---|---|---|---|---|---|---|---|---|---|---|---|---|
| | | | | | Long COVID | N | N | N | N | Y | Y | Y | Y | N | N |
| | | | | | Timepoint (months) | ac | 3 | 6 | 18 | 3 | 6 | 18 | 24 | ac | 3 |
| TRAV | TRAJ | CDR3α | TRBV | TRBJ | CDR3β | | | | | | | | | | |
| TRAV-ND | TRAV-ND | TRAV-ND | TRBV27 | TRBJ2-3 | CASSALVGAKESKDTQYF | | | | | 1 | | 1 | | | |
| TRAV-ND | TRAV-ND | TRAV-ND | TRBV27 | TRBJ2-5 | CASSPLTGLAGGKETQYF | | | | | 1 | | 1 | | | |
| TRAV-ND | TRAV-ND | TRAV-ND | TRBV7-2 | TRBJ1-1 | CASRQGSTEAFF | | | | | 1 | | | | | |
| TRAV1-2 | TRAJ27 | CAVRDWNAGKSTF | TRBV27 | TRBJ2-3 | CASSALVGAKESKDTQYF | | | | | 1 | | | | | |
| TRAV1-2 | TRAJ38 | CAVNAGNNRKLIW | TRBV4-1 | TRBJ1-1 | CASSQEWTGRTEAFF | | 1 | | | 2 | | | | | |
| TRAV20 | TRAJ31 | CAVQCHVANARLMF | TRBV27 | TRBJ2-7 | CASSHLTGGNYEQYF | | | | | 1 | | | | | |
| TRAV20 | TRAJ50 | CAVQGMKTSYDKVIF | TRBV7-9 | TRBJ1-6 | CASRSREGYNSPLHF | | | | | 1 | | | | | |
| TRAV21 | TRAJ31 | CAVMGNARLMF | TRBV27 | TRBJ2-4 | CASGFITGSQLAKNIQYF | | | | | 1 | | | | | |
| TRAV8-3 | TRAJ9 | CAVARSGTGGFKTIF | TRBV2 | TRBJ2-7 | CASSELPGPPGEQYF | | | | | 1 | | | | | |
| TRAV9-2 | TRAJ40 | CALSVTSGTYKYIF | TRBV27 | TRBJ2-5 | CASSPLTGLAGGKETQYF | | | | | 6 | 1 | 1 | | | |
| TRAV-ND | TRAV-ND | TRAV-ND | TRBV28 | TRBJ2-2 | CASSKLTGSSGELFF | | | | | | 1 | | | | |
| TRAV13-2 | TRAJ45 | GGGADGLTF | TRBV27 | TRBJ2-5 | CASSPLAGGPVRKETQYF | | | | | | 1 | | | | |
| TRAV19 | TRAJ26 | CALHPFGNFVF | TRBV28 | TRBJ2-5 | CASSALTGQAKETQYF | | | | | | 1 | | | | |
| TRAV35 | TRAJ40 | CAGPGGTYKYIF | TRBV2 | TRBJ1-2 | CASSELPVGGQGYGYTF | | | | | | 1 | | | | |
| TRAV3 | TRAJ22 | CAVRDFGSARQLTF | TRBV29-1 | TRBJ2-1 | CSVEGEDRPYNEQFF | | | | | | 1 | | | | |
| TRAV4 | TRAJ32 | CLVGDLGYGGATNKLIF | TRBV24-1/TRBV24/OR9-2 | TRBJ1-5 | CATAGANSNQPQHF | | | | | | 1 | | | | |

| | | | | | | | | | | | | | | | | | | | | | | |
|---|---|---|---|---|---|---|---|---|---|---|---|---|---|---|---|---|---|---|---|---|---|---|
| TRAV8-4 | TRAJ3 | CAVPGYSSASKIIF | TRBV20-1 | TRBJ2-7 | CSASLRGEIYEQYF |||||1||||||||||||
| TRAV13-1 | TRAJ16 | CAAFRWPERLLF | TRBV-ND | TRBV-ND | TRBV-ND ||||||1|||||||||||
| TRAV35 | TRAJ20 | CAGRPLYDYKLSF | TRBV9 | TRBJ1-1 | CASSVGEGTEAFF ||||||1|||||||||||
| TRAV8-2 | TRAJ29 | CVVILNSGNTPLVF | TRBV9 | TRBJ2-1 | CASSPYPSGGNNEQFF ||||||1|||||||||||
| TRAV8-4 | TRAJ10 | CAVSSILTGGGNKLTF | TRBV7-2 | TRBJ2-5 | CASSPRLAGQETQYF ||||||1|||||||||||
| TRAV8-4 | TRAJ44 | CAVSGPQSGTASKLTF | TRBV7-9 | TRBJ2-3 | CASSATRDYYTDTQYF ||||||1|||||||||||
| TRAV12-3 | TRAJ3 | CAMTTYSSASKIIF | TRBV27 | TRBJ2-4 | CASSQTTGPPEAKNIQYF |||||||1||||||||||
| TRAV13-2 | TRAJ37 | CAAWGSGNTGKLIF | TRBV27 | TRBJ2-4 | CASSSLVGSGSSKNIQYF |||||||1||||||||||
| TRAV-ND | TRAV-ND | TRAV-ND | TRBV19 | TRBJ1-5 | CASSIGGSFGQPQHF ||||||||1|||||||||
| TRAV-ND | TRAV-ND | TRAV-ND | TRBV25-1 | TRBJ2-3 | CASSSLGTSDPAWYF ||||||||2|||||||||
| TRAV-ND | TRAV-ND | TRAV-ND | TRBV27 | TRBJ2-4 | CASSPLTGPLVDKNIQYF ||||||||2|1||||||||
| TRAV-ND | TRAV-ND | TRAV-ND | TRBV27 | TRBJ2-5 | CASSPLTGQQEKKETQYF ||||||||2|||||||||
| TRAV-ND | TRAV-ND | TRAV-ND | TRBV29-1 | TRBJ1-1 | CSFRNTEAFF ||||||||1|||||||||
| TRAV-ND | TRAV-ND | TRAV-ND | TRBV2 | TRBJ2-6 | CASSILPLGGGANVLTF ||||||||2|||||||||
| TRAV-ND | TRAV-ND | TRAV-ND | TRBV4-1 | TRBJ1-5 | CASSQEDIRQPQHF ||||||||2|||||||||
| TRAV-ND | TRAV-ND | TRAV-ND | TRBV6-5 | TRBJ2-4 | CASSSLAGLAKNIQYF ||||||||1|||||||||
| TRAV1-1 | TRAJ36 | CAVRLGRLTGANNLLFF | TRBV11-2 | TRBJ2-3 | CASSIFGLASDTQYF ||||||||1|||||||||
| TRAV10 | TRAJ17 | CVVSAEAAGNKLTF | TRBV27 | TRBJ2-4 | CASSPLTGPLVDKNIQYF ||||||||10|||||||||
| TRAV10 | TRAJ39 | CVVSDYSNAGNMLTF | TRBV3-1/TRBV3-2 | TRBJ1-1 | CASSHRPGRPPAEAFF ||||||||1|||||||||
| TRAV19 | TRAJ13 | CALIPSGGYQKVTF | TRBV28 | TRBJ2-4 | CASSPLTGLAKNIQYF ||||||||1|||||||||
| TRAV24 | TRAJ52 | CARHAGGTSYGKLTF | TRBV6-1 | TRBJ1-1 | CASSDYFGGRENTEAFF ||||||||1|||||||||
| TRAV27 | TRAJ40 | CAGGTTRGTYKYIF | TRBV27 | TRBJ2-5 | CASSPLTGQQEKKETQYF ||||||||2|||||||||
| TRAV8-2 | TRAJ42 | CVVSGPGGSQGNLIF | TRBV2 | TRBJ1-1 | CASGRLPNTEAFF ||||||||1|||||||||
| TRAV8-6 | TRAJ49 | CAVSDGRPGNQFYF | TRBV4-1 | TRBJ1-5 | CASSQEDIRQPQHF ||||||||1|||||||||
| TRAV-ND | TRAV-ND | TRAV-ND | TRBV27 | TRBJ2-5 | CASSPLAGTGSTKETQYF | 1|||||||||||||||||
| TRAV-ND | TRAV-ND | TRAV-ND | TRBV7-8 | TRBJ2-1 | CASSMDRGRNEQFF | 1|||||||||||||||||
| TRAV14/DV4 | TRAJ44 | CAMSFTGTASKLTF | TRBV-ND | TRBV-ND | TRBV-ND | 1|||||||||||||||||
| TRAV3 | TRAJ23 | CASFLYNQGGKLIF | TRBV6-2/TRBV6-3 | TRBJ2-2 | CASMLSNTGELFF | 1|1||||||||||||||||
| TRAV-ND | TRAV-ND | TRAV-ND | TRBV14 | TRBJ2-2 | CASSQVLAGGNSGELFF ||1||||||||||||||||
| TRAV-ND | TRAV-ND | TRAV-ND | TRBV27 | TRBJ1-4 | CASSPSGSVGEKLFF ||1||||||||||||||||
| TRAV1-1 | TRAJ26 | CAGRDNYGQNFVF | TRBV27 | TRBJ2-4 | CASSPLTGGPIAKNIQYF ||1||||||||||||||||
| TRAV1-1 | TRAJ8 | CAVRDRGMNTGFQKLVF | TRBV4-1 | TRBJ2-1 | CASSQDLGTAYNEQFF ||1||||||||||||||||
| TRAV1-2 | TRAJ31 | CAVRGYNNARLMF | TRBV-ND | TRBV-ND | TRBV-ND ||1||||||||||||||||
| TRAV1-2 | TRAJ38 | CAANAGNNRKYIW | TRBV4-1 | TRBJ1-1 | CASSQEWTGRTEAFF ||1||||||||||||||||
| TRAV1-2 | TRAJ39 | CAVREAGNMLTF | TRBV2 | TRBJ2-1 | CASSALVDRREQFF ||1||||||||||||||||
| TRAV16 | TRAJ40 | CALSKRNSGTYKYIF | TRBV27 | TRBJ2-5 | CASSPLTGLAGEKETQYF ||1||||||||||||||||
| TRAV17 | TRAJ23 | CATGYYNQGGKLIF | TRBV27 | TRBJ2-4 | CASSFLTGPPQNKNIQYF ||1|1|||||||||||||||
| TRAV21 | TRAJ13 | CAAWGYQKVTF | TRBV27 | TRBJ2-4 | CASSPLAGGPEVKNIQYF ||3||||||||||||||||
| TRAV24 | TRAJ9 | CAPPVGFKTIF | TRBV15 | TRBJ2-2 | CATRVELNTGELFF ||2||||||||||||||||
| TRAV27 | TRAJ21 | CAGADFNKFYF | TRBV2 | TRBJ2-1 | CASTPGLAGATSYNEQFF ||1||||||||||||||||
| TRAV27 | TRAJ45 | CAGAMYSGGGADGLTF | TRBV4-1 | TRBJ2-7 | CASSQDWGTASRGVQYF ||2||||||||||||||||
| TRAV29/DV5 | TRAJ13 | CAVSPGRGGYQKVTF | TRBV28 | TRBJ1-1 | CASSSMTGVNTEAFF ||1||||||||||||||||
| TRAV38-1 | TRAJ49 | CAFMKLDQFYF | TRBV27 | TRBJ2-7 | CASSLSASGWREQYF ||1||||||||||||||||
| TRAV8-1 | TRAJ27 | CAFWGEGKSTF | TRBV27 | TRBJ2-7 | CASSSLTSGSWSYEQYF ||1||||||||||||||||
| TRAV8-1 | TRAJ7 | CAVMGSGRLAF | TRBV27 | TRBJ2-4 | CASSYITGLAKNIQYF ||1||||||||||||||||
| TRAV8-2/TRAV8-4 | TRAJ47 | CVGTTYGNKLVF | TRBV20-1 | TRBJ2-1 | CSARDPGGAGGSEQFF ||1||||||||||||||||
| TRAV-ND | TRAV-ND | TRAV-ND | TRBV28 | TRBJ1-1 | CASSSMTGVNTEAFF |||1|||||||||||||||
| TRAV4 | TRAJ4 | CLVGDPVGYNKLIF | TRBV27 | TRBJ2-7 | CASSYAGTSGRFYEQYF |||1|||||||||||||||
| TRAV-ND | TRAV-ND | TRAV-ND | TRBV27 | TRBJ2-1 | CASSSGLAGGAFNEQFF ||||1||||||||||||||
| TRAV-ND | TRAV-ND | TRAV-ND | TRBV28 | TRBJ1-5 | CASSPLTGGFGNQPQHF ||||1||||||||||||||
| TRAV1-2 | TRAJ16 | CAVRPLSDGQKLLF | TRBV27 | TRBJ2-4 | CASSPVTGTLLAKNIQYF ||||1||||||||||||||
| TRAV9-2 | TRAJ40 | CALSASTSGTYKYIF | TRBV27 | TRBJ2-5 | CASSPLTGSGMLKETQYF ||||1||||||||||||||
| DPB4/S$_{167}$ | | | | | Donor | 10 | 10 | 10 | 13 | 13 | 13 | 13 | 13 | 6 | 6 | 3 | 15 | 15 | 15 | 14 | 14 | 14 |
| | | | | | Long COVID | Y | Y | Y | Y | Y | Y | Y | Y | Y | Y | Y | N | N | N | Y | Y | Y |
| | | | | | Timepoint (months) | ac | 6 | 12 | ac | 3 | 6 | 12 | 18 | ac | 6 | 3 | ac | 6 | 18 | 3 | 6 | 24 |
| TRAV | TRAJ | CDR3α | TRBV | TRBJ | CDR3β |||||||||||||||||||
| TRAV35 | TRAJ42 | CAGLNYGGSQGNLIF | TRBV-ND | TRBV-ND | TRBV-ND |||||||||||1||||||||
| TRAV35 | TRAJ44 | CAGYLFTGTASKLTF | TRBV30 | TRBJ1-2 | CAWRSIGYTF |||||||||1||||||||||
| TRAV-ND | TRAV-ND | TRAV-ND | TRBV7-2/TRBV7-8 | TRBJ1-2 | CASSPRTGGSGYTF ||||||||||1|||||||||
| TRAV-ND | TRAV-ND | TRAV-ND | TRBV7-2/TRBV7-8 | TRBJ2-2 | CASSPRLAGATGELFF ||||||||||1|||||||||
| TRAV35 | TRAJ42 | CAALNYGGSQGNLIF | TRBV28 | TRBJ1-2 | CASSKNRGFGYTF ||||||||||1|||||||||
| TRAV35 | TRAJ42 | CAGLNYGGSQGNLIF | TRBV19 | TRBJ1-2 | CASSYRPGSLYGYTF ||||||||||1|||||||||
| TRAV35 | TRAJ42 | CAGLNYGGSQGNLIF | TRBV28 | TRBJ1-2 | CASSGRGGWGYTF ||||||||||1|||||||||
| TRAV35 | TRAJ42 | CAGQLYGGSQGNLIF | TRBV5-5 | TRBJ1-2 | CASSGTGGLGYTF ||||||||||1|||||||||
| TRAV35 | TRAJ42 | CAGQNYGGSQGNLIF | TRBV7-2/TRBV7-8 | TRBJ2-2 | CASSLGWPSNTGELFF ||||||||||1|||||||||
| TRAV35 | TRAJ42 | CALMNYGGSQGNLIF | TRBV3-1/TRBV3-2 | TRBJ1-2 | CASSPRGAWGYTF ||||||||||1|||||||||
| TRAV-ND | TRAV-ND | TRAV-ND | TRBV10-3 | TRBJ1-2 | CAISESKNGLYGYTF | 1|||||||||||||||||
| TRAV-ND | TRAV-ND | TRAV-ND | TRBV27 | TRBJ2-1 | CASSFRQGAFEQFF | 1|||||||||||||||||
| TRAV-ND | TRAV-ND | TRAV-ND | TRBV9 | TRBJ1-2 | CASSVRGSGGYTF | 1|||||||||||||||||
| TRAV35 | TRAJ42/TRAJ53 | CAALNSGGSNYKLTF | TRBV5-5 | TRBJ2-1 | CASSPRLAGASYNEQFF | 1|||||||||||||||||
| TRAV35 | TRAJ42 | CAARNYGGSQGNLIF | TRBV6-4 | TRBJ1-2 | CASSDSFGYTF | 5|1|1|||||||||||||||
| TRAV35 | TRAJ42 | CAGLNYGGSQGNLIF | TRBV11-2 | TRBJ2-2 | CASSPRRGVPTGELFF | 1|||||||||||||||||
| TRAV35 | TRAJ42 | CAGLNYGGSQGNLIF | TRBV5-5 | TRBJ2-1 | CASSPRLAGASYNEQFF | 4|||||||||||||||||
| TRAV35 | TRAJ42 | CAGMNYGGSQGNLIF | TRBV7-9 | TRBJ1-2 | CASTQGTFGYTF | 1||1|||||||||||||||
| TRAV35 | TRAJ42 | CAGQLYGGSQGNLIF | TRBV7-9 | TRBJ1-2 | CASSLRGAGGYTF | 1|1|1|||||||||||||||
| TRAV35 | TRAJ42 | CAGQNYGGSQGNLIF | TRBV12-3/TRBV12-4 | TRBJ1-2 | CASSFRQGALDGYTF | 1|||||||||||||||||
| TRAV35 | TRAJ42 | CAGQNYGGSQGNLIF | TRBV18 | TRBJ1-2 | CASSPRPDPMGYTF | 1|||||||||||||||||
| TRAV35 | TRAJ42 | CAGQNYGGSQGNLIF | TRBV6-5 | TRBJ1-2 | CASSYRQGSIYGYTF | 1|||||||||||||||||
| TRAV35 | TRAJ42 | CAGRNYGGSQGNLIF | TRBV14 | TRBJ1-2 | CASSQGQGWEYGYTF | 1|||||||||||||||||
| TRAV-ND | TRAV-ND | TRAV-ND | TRBV30 | TRBJ1-2 | CAWALRTGSLYGYTF ||1||||||||||||||||
| TRAV35 | TRAJ42 | CAGLNYGGSQGNLIF | TRBV14 | TRBJ1-1 | CASSHRAVGASVNEAFF ||3||||||||||||||||
| TRAV35 | TRAJ42 | CAGLNYGGSQGNLIF | TRBV28 | TRBJ1-2 | CASTLNRGGLYGYTF ||1||||||||||||||||
| TRAV35 | TRAJ42 | CAGMNYGGSQGNLIF | TRBV-ND | TRBV-ND | TRBV-ND ||1||||1||||||||||||
| TRAV35 | TRAJ42 | CAGMNYGGSQGNLIF | TRBV7-2 | TRBJ2-5 | CASTLRPTGLETQYF ||1|1|||||||||||||||
| TRAV41 | TRAJ49 | CAGVTGNQFYF | TRBV27 | TRBJ1-2 | CASSYRQGALSGYTF ||1||||||||||||||||
| TRAV-ND | TRAV-ND | TRAV-ND | TRBV15 | TRBJ1-2 | CATSSRGGSLYGYTF |||1|||||||||||||||
| TRAV35 | TRAJ42 | CAAQNYGGSQGNLIF | TRBV-ND | TRBV-ND | TRBV-ND |||1|||||||||||||||
| TRAV35 | TRAJ42 | CAGFNYGGSQGNLIF | TRBV20-1/TRBV20/OR9-2 | TRBJ2-1 | CSARKGQGIGEQFF |||1|||||||||||||||
| TRAV35 | TRAJ42 | CAGLNYGGSQGNLIF | TRBV12-3/TRBV12-4 | TRBJ1-2 | CASSRRQGNIYGYTF |||1|||||||||||||||
| TRAV35 | TRAJ42 | CAGLNYGGSQGNLIF | TRBV19 | TRBJ1-2 | CASTRPGALYGYTF |||2|||||||||||||||
| TRAV35 | TRAJ42 | CAGLNYGGSQGNLIF | TRBV28 | TRBJ1-2 | CASTLSRGALYGYTF |||2|||||||||||||||
| TRAV35 | TRAJ42 | CAGQNYGGSQGNLIF | TRBV-ND | TRBV-ND | TRBV-ND |||1|||||||||||||||
| TRAV35 | TRAJ42 | CAGQNYGGSQGNLIF | TRBV7-2 | TRBJ2-2 | CASSSARAGGPNTGELFF |||1|||||||||||||||
| TRAV35 | TRAJ53 | CAGLNSGGSNYKLTF | TRBV6-4 | TRBJ1-2 | CASSPRQGPPAGYTF |||2|||||||||||||||
| TRAV-ND | TRAV-ND | TRAV-ND | TRBV30 | TRBJ1-2 | CACRPRQGLGGYTF ||||1||||||||||||||
| TRAV-ND | TRAV-ND | TRAV-ND | TRBV30 | TRBJ1-2 | CAWKEGLGYTF ||||1||||||||||||||
| TRAV35 | TRAJ17 | CAGGLIKAAGNKLTF | TRBV4-1 | TRBJ2-7 | CASSQGEAGKQYF ||||1||||||||||||||
| TRAV35 | TRAJ17 | CAGQLYKAAGNKLTF | TRBV-ND | TRBV-ND | TRBV-ND ||||1|1|||||||||||||
| TRAV35 | TRAJ42 | CAGFNYGGSQGNLIF | TRBV4-1 | TRBJ1-2 | CASSQSFSYTF ||||1||||||||||||||
| TRAV35 | TRAJ42 | CAGLIYGGSQGNLIF | TRBV3-1/TRBV3-2 | TRBJ1-2 | CASSHRGSLYGYTF ||||1||||||||||||||
| TRAV35 | TRAJ42 | CAGLNYGGSQGNLIF | TRBV4-1 | TRBJ1-2 | CASSQSMGYTF ||||1||||||||||||||
| TRAV35 | TRAJ42 | CAGLNYGGSQGNLIF | TRBV7-9 | TRBJ1-2 | CASSSRTGTLYGYTF ||||1||||||||||||||
| TRAV35 | TRAJ42 | CAGMNYGGSQGNLIF | TRBV25-1 | TRBJ1-2 | CASSARGANGYTF ||||1||||||||||||||
| TRAV35 | TRAJ42 | CAGQNYGGSQGNLIF | TRBV9 | TRBJ1-2 | CASSPRDRTLGYTF ||||1||||||||||||||
| TRAV35 | TRAJ42 | CAVLNYGGSQGNLIF | TRBV19 | TRBJ1-2 | CASSTSFGYTF ||||1||||||||||||||
| TRAV40 | TRAJ56 | CLLEFNRYTGAHSKLTF | TRBV11-2 | TRBJ1-2 | CASSPRGDSLWGYTF ||||1||||||||||||||
| TRAV40 | TRAJ56 | CLLEFYRYTGANSKLTF | TRBV11-2 | TRBJ1-2 | CASSPRGDSLWGYTF ||||1||||||||||||||
| TRAV8-4 | TRAJ42 | CAVSDVIDYGGSQGNLIF | TRBV9 | TRBJ1-2 | CASSTRDSRPGTF ||||1||||||||||||||

| | | | | | | | | | | | | | | | | | | |
|---|---|---|---|---|---|---|---|---|---|---|---|---|---|---|---|---|---|---|
| TRAV-ND | TRAV-ND | TRAV-ND | TRBV11-2 | TRBJ1-2 | CASSPRGDSLWGYTF | 1 | | | | | | | | | | | | |
| TRAV-ND | TRAV-ND | TRAV-ND | TRBV20-1/TRBV20/OR9-2 | TRBJ1-1 | CSARDFRGAATEAFF | 1 | | | | | | | | | | | | |
| TRAV-ND | TRAV-ND | TRAV-ND | TRBV25-1 | TRBJ2-7 | CASSDRGLLGQYF | 1 | | | | | | | | | | | | |
| TRAV-ND | TRAV-ND | TRAV-ND | TRBV30 | TRBJ1-2 | CAHTRGVGYTF | 1 | | | | | | | | | | | | |
| TRAV35 | TRAJ17 | CAGQLFKAAGNKLTF | TRBV-ND | TRBV-ND | TRBV-ND | 1 | | | | | | | | | | | | |
| TRAV35 | TRAJ42 | CAGLLYGGSQGNLIF | TRBV9 | TRBJ2-2 | CASSLRSDPGGTGELFF | 1 | | | | | | | | | | | | |
| TRAV35 | TRAJ42 | CAGLNYGGSQGNLIF | TRBV20-1/TRBV20/OR9-2 | TRBJ1-2 | CSARTGVGYTF | 1 | | | | | | | | | | | | |
| TRAV35 | TRAJ42 | CAGRNYGGSQGNLIF | TRBV11-2 | TRBJ2-3 | CASSLRGDTQYF | 1 | | | | | | | | | | | | |
| TRAV35 | TRAJ53 | CAGFNSGGSNYKLTF | TRBV15 | TRBJ1-2 | CATSRDRAPGPHGYTF | 1 | | 1 | | | | | | | | | | |
| TRAV-ND | TRAV-ND | TRAV-ND | TRBV14 | TRBJ1-2 | CASSPARGLGGYTF | | 1 | | | | | | | | | | | |
| TRAV35 | TRAJ17 | CAGQLYKAAGNKLTF | TRBV11-2 | TRBJ1-2 | CASSPREGTGYGYTF | | 1 | | | | | | | | | | | |
| TRAV35 | TRAJ42 | CAGLNYGGSQGNLIF | TRBV9 | TRBJ1-3 | CASSVGRDRFFSGNTIYF | | 1 | | | | | | | | | | | |
| TRAV35 | TRAJ42 | CAGMNYGGSQGNLIF | TRBV10-3 | TRBJ1-2 | CAMQVGVGYTF | | 1 | | | | | | | | | | | |
| TRAV35 | TRAJ42 | CAGMNYGGSQGNLIF | TRBV18 | TRBJ1-2 | CASSPSTGSPYGYTF | | 1 | | | | | | | | | | | |
| TRAV35 | TRAJ42 | CAGQLYGGSQGNLIF | TRBV30 | TRBJ1-2 | CATAKGVGYTF | | 1 | | | | | | | | | | | |
| TRAV35 | TRAJ42 | CAGQNYGGSQGNLIF | TRBV4-1 | TRBJ1-2 | CASRSGIGYTF | | 2 | | | | | | | | | | | |
| TRAV35 | TRAJ42 | CAGQNYGGSQGNLIF | TRBV5-4 | TRBJ2-2 | CASSTGTSGGINTGELFF | | 1 | | | | | | | | | | | |
| TRAV35 | TRAJ42 | CAGQNYGGSQGNLIF | TRBV9 | TRBJ2-5 | CASSPRGQGPGETQYF | | 1 | | | | | | | | | | | |
| TRAV-ND | TRAV-ND | TRAV-ND | TRBV19 | TRBJ2-5 | CASSTDRGLQTQYF | | | 1 | | | | | | | | | | |
| TRAV35 | TRAJ42 | CAGLNYGGSQGNLIF | TRBV12-3/TRBV12-4 | TRBJ2-2 | CASSPRLAGATGELFF | | | 1 | | | | | | | | | | |
| TRAV35 | TRAJ42 | CAGQLYGGSQGNLIF | TRBV30 | TRBJ1-2 | CAWGKSLGYTF | | | 1 | | | | | | | | | | |
| TRAV35 | TRAJ42 | CAGVNYGGSQGNLIF | TRBV5-5 | TRBJ1-2 | CASSPGTGLGYTF | | | 1 | | | | | | | | | | |
| TRAV35 | TRAJ42 | CAYMNYGGSQGNLIF | TRBV3-1/TRBV3-2 | TRBJ1-2 | CASRPQQSTLHGYTF | | | 1 | | | | | | | | | | |
| TRAV35 | TRAJ53 | CAALNSGGSNYKLTF | TRBV15 | TRBJ1-2 | CATSRERTGGVSGYTF | | | 1 | | | | | | | | | | |
| TRAV-ND | TRAV-ND | TRAV-ND | TRBV11-3 | TRBJ1-2 | CASSARGAHGYTF | | | | 1 | | | | | | | | | |
| TRAV35 | TRAJ42 | CAALNYGGSQGNLIF | TRBV30 | TRBJ1-2 | CATRSGVGYTF | | | | 1 | | | | | | | | | |
| TRAV-ND | TRAV-ND | TRAV-ND | TRBV10-3 | TRBJ1-2 | CAISEGVGYTF | | | | | | | | | | | 1 | | |
| TRAV-ND | TRAV-ND | TRAV-ND | TRBV11-3 | TRBJ2-3 | CASSRRGDTQYF | | | | | | | | | | | 1 | | |
| TRAV-ND | TRAV-ND | TRAV-ND | TRBV19 | TRBJ1-2 | CASSPKGAVYGYTF | | | | | | | | | | | 1 | | |
| TRAV-ND | TRAV-ND | TRAV-ND | TRBV19 | TRBJ1-2 | CAVKQGAFGYTF | | | | | | | | | | | 1 | | |
| TRAV-ND | TRAV-ND | TRAV-ND | TRBV27 | TRBJ2-2 | CASSPARSANTGELFF | | | | | | | | | | | 2 | | |
| TRAV-ND | TRAV-ND | TRAV-ND | TRBV6-4 | TRBJ1-2 | CASPGPPCGYTF | | | | | | | | | | | 1 | | |
| TRAV34 | TRAJ44 | CGIRGTASKLTF | TRBV9 | TRBJ2-2 | CASSPRDRANTGELFF | | | | | | | | | | | 1 | | |
| TRAV35 | TRAJ17 | CAGQIYKAAGNKLTF | TRBV29-1 | TRBJ1-2 | CSVGTGSPHGYTF | | | | | | | | | | | 1 | 1 | |
| TRAV35 | TRAJ17 | CAGQLNKAAGNKLTF | TRBV20-1 | TRBJ1-2 | CSARTGVGYTF | | | | | | | | | | | 1 | | |
| TRAV35 | TRAJ17 | CAGQLNKAAGNKLTF | TRBV7-2 | TRBJ1-2 | CASSLTGVYGYTF | | | | | | | | | | | 1 | | |
| TRAV35 | TRAJ17 | CAGQMFQRCRNKLIF | TRBV19 | TRBJ2-7 | CASSPRGDEQYF | | | | | | | | | | | 1 | | |
| TRAV35 | TRAJ42 | CAALNYGGSQGNLIF | TRBV10-1 | TRBJ2-3 | CASGTSLAQYF | | | | | | | | | | | 1 | 1 | |
| TRAV35 | TRAJ42 | CAGKNYGGSQGNLIF | TRBV11-3 | TRBJ2-3 | CASSRRGDTQYF | | | | | | | | | | | 2 | | |
| TRAV35 | TRAJ42 | CAGLNYGGSQGNLIF | TRBV19 | TRBJ1-2 | CASRPGTFGYTF | | | | | | | | | | | 1 | | |
| TRAV35 | TRAJ42 | CAGLNYGGSQGNLIF | TRBV19 | TRBJ1-5 | CASTPDRGLRAQHF | | | | | | | | | | | 1 | | |
| TRAV35 | TRAJ42 | CAGLNYGGSQGNLIF | TRBV27 | TRBJ2-2 | CASSPARSANTGELFF | | | | | | | | | | | 2 | | |
| TRAV35 | TRAJ42 | CAGMNYGGSQGNLIF | TRBV7-2 | TRBJ2-7 | CASGPPSGLRQYF | | | | | | | | | | | 1 | | |
| TRAV35 | TRAJ42 | CAGQNYGGSQGNLIF | TRBV12-3/TRBV12-4 | TRBJ1-2 | CASSSSPGSLYGYTF | | | | | | | | | | | 1 | | |
| TRAV36/DV7 | TRAJ30 | CADGDDKIIF | TRBV19 | TRBJ2-3 | CASSIVRGHNTDTQYF | | | | | | | | | | | 1 | | |
| TRAV6 | TRAJ31 | CALMGARLMF | TRBV-ND | TRBV-ND | TRBV-ND | | | | | | | | | | | 1 | | |
| TRAV8-6 | TRAJ29 | CAVAKGNTPLVF | TRBV28 | TRBJ2-7 | CASRSAGTSYEQYF | | | | | | | | | | | 1 | | |
| TRAV-ND | TRAV-ND | TRAV-ND | TRBV9 | TRBJ2-2 | CASSPRDRANTGELFF | | | | | | | | | | | | 1 | |
| TRAV35 | TRAJ42 | CAALNYGGSQGNLIF | TRBV5-4 | TRBJ2-2 | CASSSSGLLELFF | | | | | | | | | | | | 1 | |
| TRAV35 | TRAJ42 | CAGLNYGGSQGNLIF | TRBV3-1/TRBV3-2 | TRBJ1-2 | CATRGGIGYTF | | | | | | | | | | | | 1 | |
| TRAV35 | TRAJ42 | CAGQNYGGSQGNLIF | TRBV4-3 | TRBJ2-2 | CASSQVVPTNTGELFF | | | | | | | | | | | | 1 | |
| TRAV35 | TRAJ42 | CAGRNYGGSQGNLIF | TRBV19 | TRBJ1-2 | CASSMAFGYTF | | | | | | | | | | | | 1 | |
| TRAV35 | TRAJ53 | CAGFNSGGSNYKLTF | TRBV14 | TRBJ2-2 | CASSPLRGVGELFF | | | | | | | | | | | | 1 | |
| TRAV-ND | TRAV-ND | TRAV-ND | TRBV19 | TRBJ1-2 | CASSMRGALGYTF | | | | | | | | | | | | | 1 |
| TRAV35 | TRAJ42 | CAALNYGGSQGNLIF | TRBV18 | TRBJ2-7 | CASSPYPFSGTNEQYF | | | | | | | | 1 | | | | | |
| TRAV-ND | TRAV-ND | TRAV-ND | TRBV15 | TRBJ1-2 | CATSAGTGSLDGYTF | | | | | | | | | 1 | | | | |
| TRAV-ND | TRAV-ND | TRAV-ND | TRBV27 | TRBJ2-2 | CASSFRRVGTGELFF | | | | | | | | | 1 | | | | |
| TRAV-ND | TRAV-ND | TRAV-ND | TRBV2 | TRBJ2-1 | CASSALVNRREQFF | | | | | | | | | 1 | | | | |
| TRAV-ND | TRAV-ND | TRAV-ND | TRBV30 | TRBJ1-2 | CARRTGGLGGYTF | | | | | | | | | 1 | | | | |
| TRAV-ND | TRAV-ND | TRAV-ND | TRBV7-2 | TRBJ2-3 | CASSLRGRDLAGEGTDTQYF | | | | | | | | | 1 | | | | |
| TRAV14/DV4 | TRAJ44 | CAMRESGGASKLTF | TRBV5-1 | TRBJ2-1 | CASSLAGAYEQFF | | | | | | | | | 1 | | | | |
| TRAV20 | TRAJ48 | CAVPVFGGAGNEKLTF | TRBV9 | TRBJ1-2 | CASSNNRGQGYTF | | | | | | | | | 1 | | | | |
| TRAV25 | TRAJ36 | CAGTQTGANNLFF | TRBV2 | TRBJ2-3 | CASRRWTSGGGTDTQYF | | | | | | | | | 1 | | | | |
| TRAV35 | TRAJ17 | CAGQLHKAAGNKLTF | TRBV6-5 | TRBJ2-6 | CASSYSVRGGGANVLTF | | | | | | | | | 1 | | | | |
| TRAV35 | TRAJ42 | CAGRLYGGSQGNLIF | TRBV-ND | TRBV-ND | TRBV-ND | | | | | | | | | 1 | | | | |
| TRAV35 | TRAJ42 | CAGRNYGGSQGNLIF | TRBV6-2/TRBV6-3 | TRBJ1-2 | CASSYSFGYTF | | | | | | | | | 1 | | | | |
| TRAV-ND | TRAV-ND | TRAV-ND | TRBV10-2 | TRBJ1-2 | CASSERGQLYGYTF | | | | | | | | | | 1 | | | |
| TRAV-ND | TRAV-ND | TRAV-ND | TRBV11-2 | TRBJ1-2 | CASSFRGDYGYTF | | | | | | | | | | 1 | | | |
| TRAV-ND | TRAV-ND | TRAV-ND | TRBV2 | TRBJ1-2 | CASSVRQGPLYGYTF | | | | | | | | | | 1 | | | |
| TRAV35 | TRAJ42 | CAGLNYGGSQGNLIF | TRBV24-1/TRBV24/OR9-2 | TRBJ2-7 | CATSATSGIQEQYF | | | | | | | | | | 1 | | | |
| TRAV35 | TRAJ42 | CAGMNYGGSQGNLIF | TRBV24-1/TRBV24/OR9-2 | TRBJ1-2 | CATSRRGGDGYTF | | | | | | | | | | 1 | | | |
| TRAV35 | TRAJ42 | CAGQNYGGSQGNLIF | TRBV6-4 | TRBJ2-2 | CASSDSGPTRTGELFF | | | | | | | | | | 1 | | | |
| TRAV35 | TRAJ42 | CAVLNYGGSQGNLIF | TRBV28 | TRBJ2-5 | CASTGGRGLKTQYF | | | | | | | | | | 1 | | | |

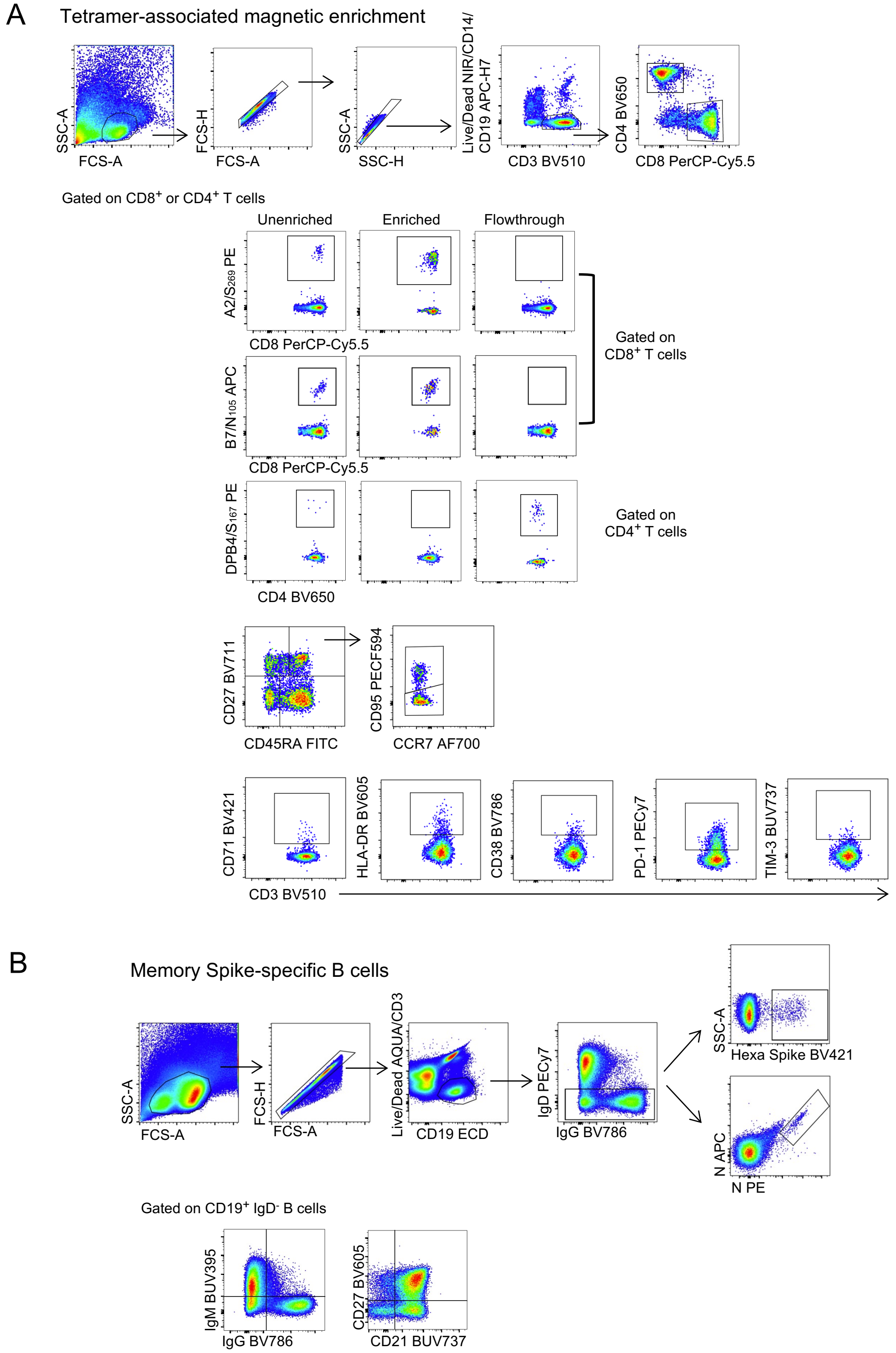


**Fig. S1.** Gating strategy of TAME and probe assays. Representative gating strategy for (*A*) measuring enriched tetramer-specific CD8$^{+}$ and CD4$^{+}$ T cells and (*B*) measuring *ex vivo* probe-specific B cells.

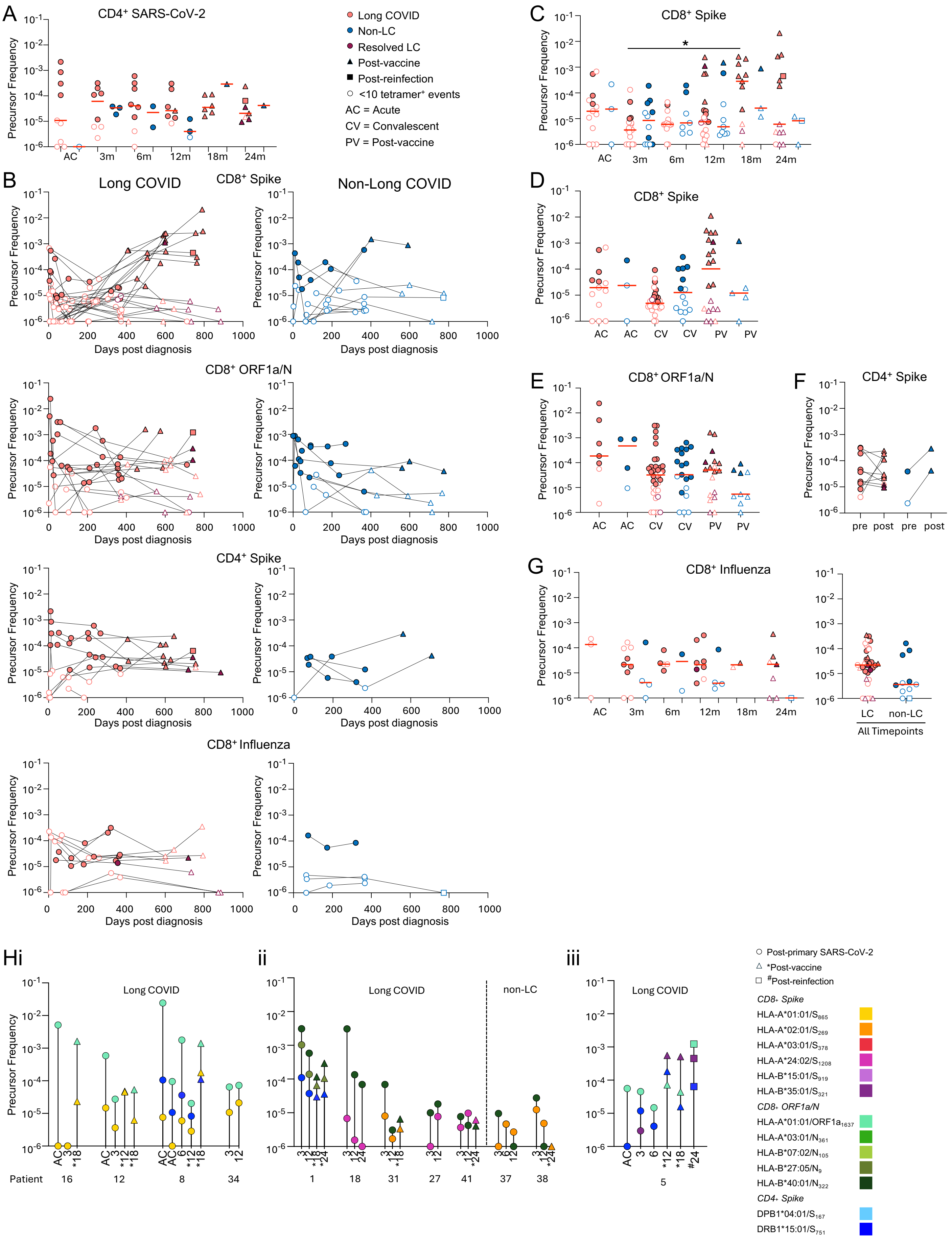


**Fig. S2**. Legend on following page

**Fig. S2**. Longitudinal analyses of antigen-specific T cell responses. (*A*) $CD4^+$ SARS-CoV-2-specific $tetramer^+$ T cell frequencies in people with long COVID and non-LC controls over time post diagnosis. (*B*) Frequency of $CD8^+$ spike-specific, $CD8^+$ ORF1a/N-specific, $CD4^+$ spike-specific and $CD8^+$ influenza-specific $tetramer^+$ T cells from people with long COVID and non-LC controls graphed longitudinally. (*C*) Frequency of $CD8^+$ spike-specific $tetramer^+$ over time and (*D*) grouped by acute, convalescent and post-COVID-19 vaccination with repeated measures per epitope (i.e. donor with multiple samples within a grouping) averaged. (*E*) $CD8^+$ ORF1a/N-specific $tetramer^+$ T cell frequencies group by acute, convalescent and post-COVID-19 vaccination. (*F*) Frequency of spike-specific $CD4^+$ T cells pre- and post- COVID-19 vaccination. (*G*) Frequency of $CD8^+$ influenza-specific $tetramer^+$ T cells ($A2/M1_{58}$, $A24/PB1_{498}$ and $B35/NP_{418}$) at binned timepoints and with timepoints combined. (*H*) SARS-CoV-2 epitope-specific T cells in longitudinal individuals (>3 months) graphed per individual per timepoint showing frequencies in (*i*) individuals with $A1/ORF1a_{1637}$ and $A1/S_{865}$, (*ii*) individuals with $HLA\text{-}B40/N_{322}$ and a second $CD8^+$ SARS-CoV-2 epitope, (*iii*) reinfected long COVID donor#5. Statistical significance determined by Dunn's multiple comparison test across study timepoints and vaccination groups (*A, C-E, G*) and Wilcoxon matched-pairs sign rank test comparing pre and post vaccination (*F*). The frequency of $tetramer^+$ cells have been shifted up by $10^{-6}$ (i.e. no detected $tetramer^+$ events displayed as $10^{-6}$) to allow for visibility on the logarithmic y axis. Any samples with <10 $tetramer^+$ events are shown as open symbols. 7 datapoints are from previously described COVID-19 adult cohort (2).

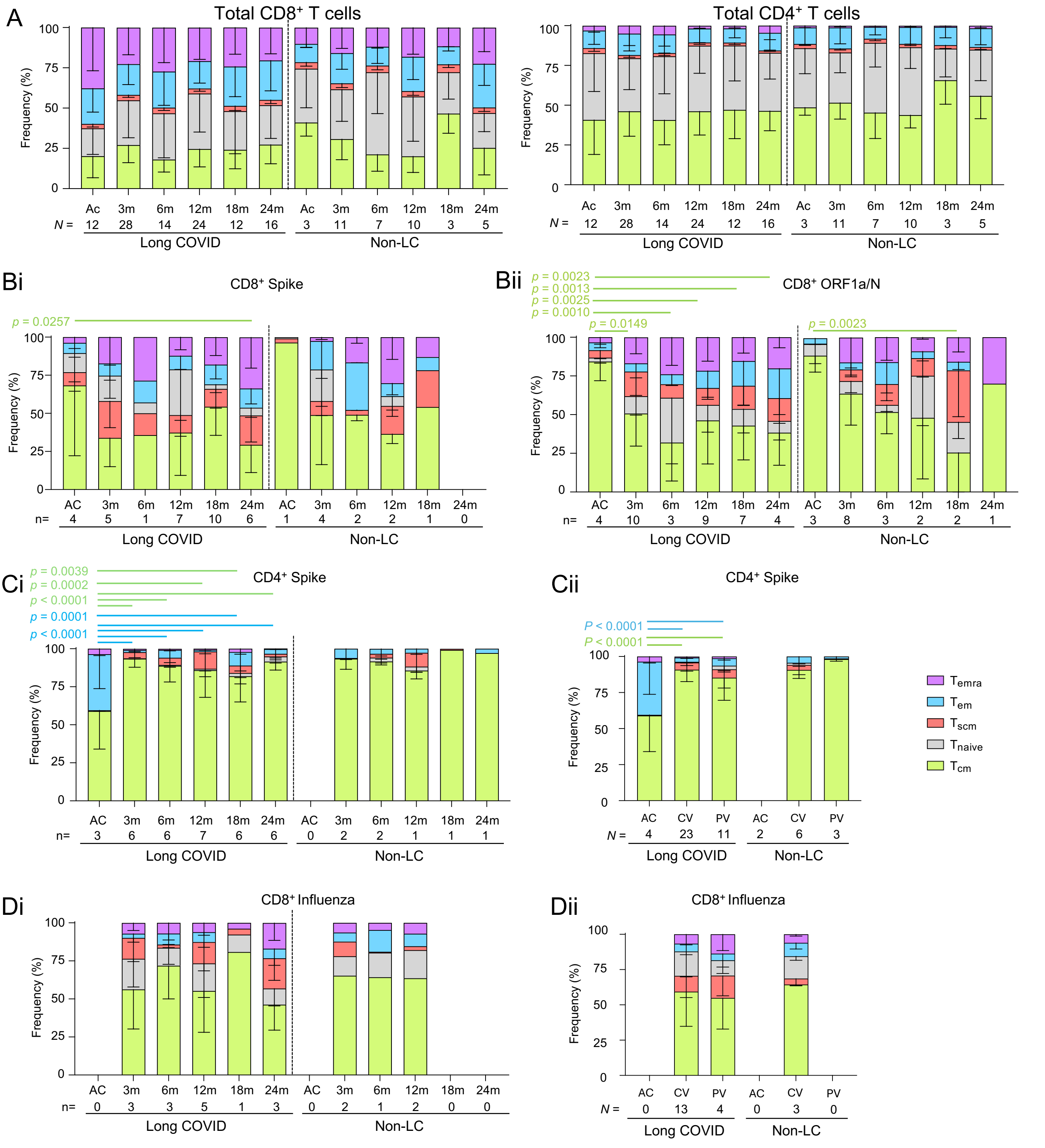


**Fig. S3.** Expanded phenotypic analysis of SARS-CoV-2-specific tetramer⁺ T cells. Phenotypic analysis of T cells in people with long COVID and non-LC controls showing (*A*) total unenriched CD8⁺ and CD4⁺ T cells across the study timepoints, (*Bi*) enriched CD8⁺ spike-specific and (*Bii*) ORF1a/N-specific tetramer⁺ T cells across binned timepoints and (*C*) enriched CD4⁺ spike-specific and (*D*) enriched CD8⁺ influenza-specific tetramer⁺ T cells across (*i*) binned and (*ii*) pooled timepoints. Statistical significance determined by Tukey's multiple comparison's test. Only samples with 10 or more tetramer⁺ events are included in the phenotypic analysis. 7 datapoints are derived from our previous COVID-19 adult cohort (2).

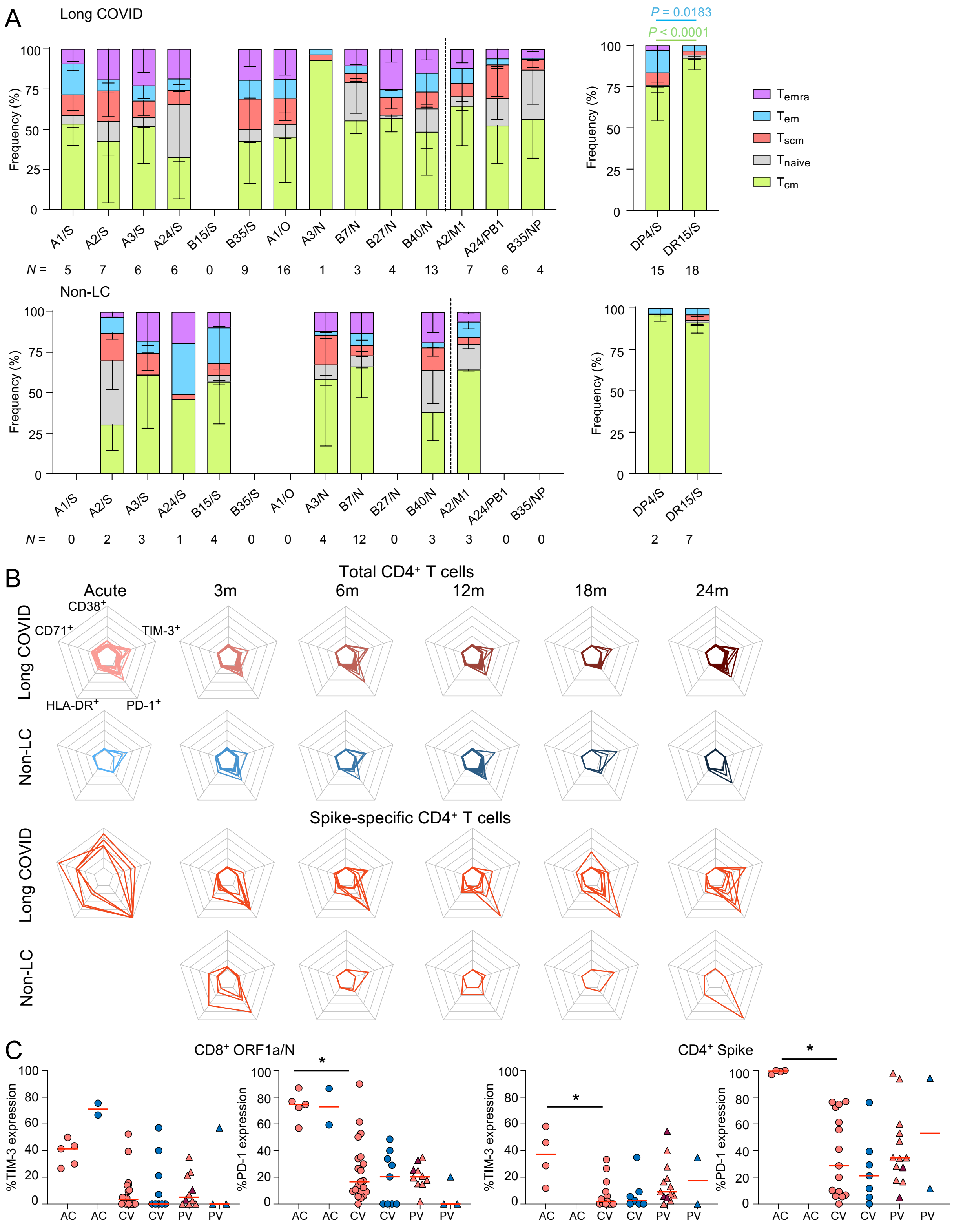


**Fig. S4.** Phenotypic analysis across epitopes. (*A*) Phenotypic analysis of T cells in people with long COVID and non-LC controls showing per epitope with all timepoints combined. 7 datapoints are derived from our previous COVID-19 adult cohort (41). (*B*) Radar plots of activation markers (HLA-DR$^+$, CD71$^+$, CD38$^+$, TIM-3$^+$ and PD-1$^+$) of total CD4$^+$ and spike-specific CD4$^+$ T cells in long COVID and non-LC groups. Radar shows frequency of expression from 0% (center) to 100% (outer edge). (*C*) TIM-3 and PD-1 expression of ORF1a/N-specific CD8$^+$ and spike-specific CD4$^+$ T cells in long COVID and non-LC groups. Statistical significance determined by (*A*) Tukey's multiple comparison's test and (*B*) Dunn's multiple comparison test. Only samples with 10 or more tetramer$^+$ events are included in the phenotypic analysis.

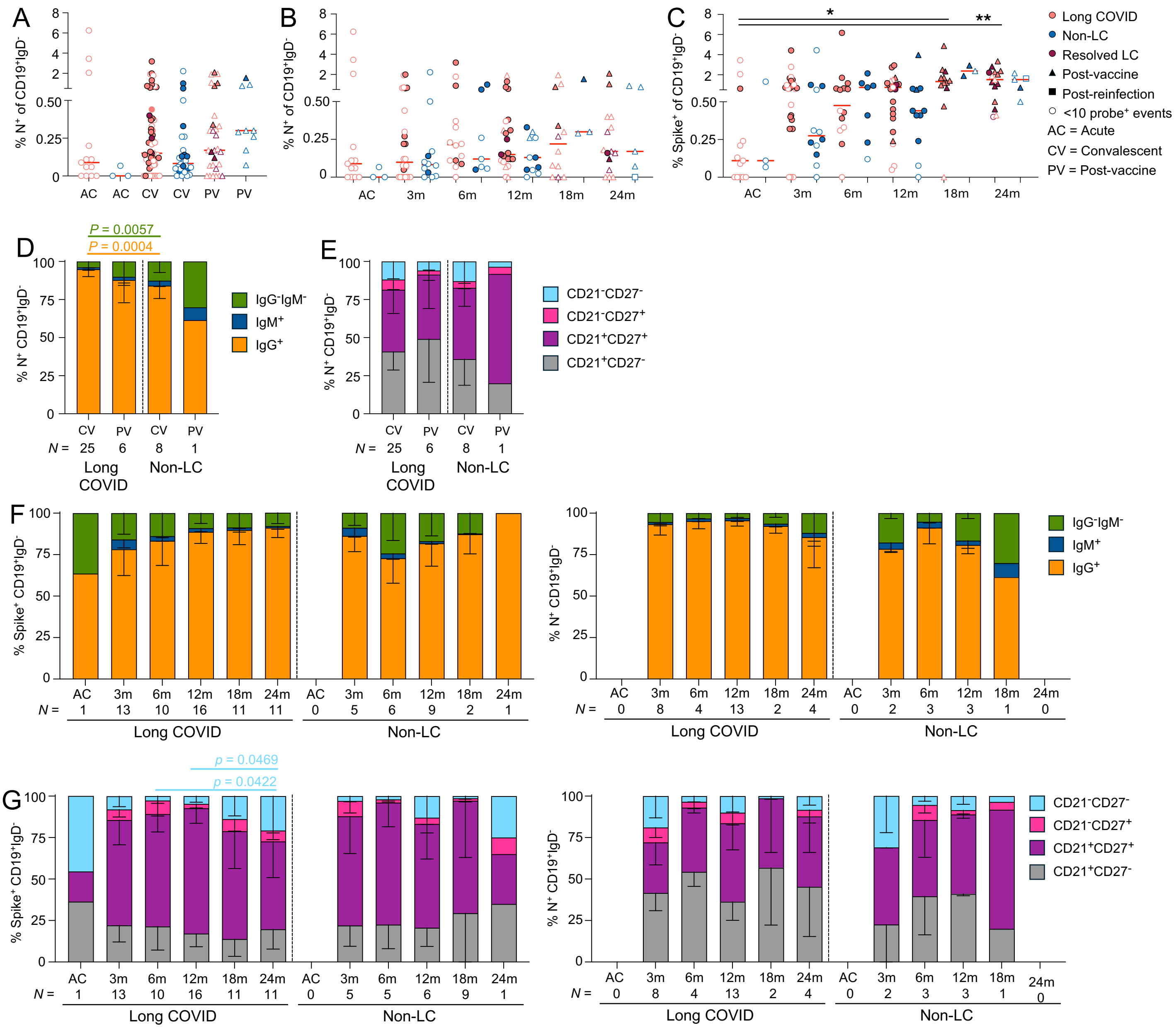


**Fig. S5.** Expanded phenotypic analysis of SARS-CoV-2 specific B cells. (*A*) Frequency of SARS-CoV-2 N-specific probe⁺ B cells in people with long COVID and non-LC controls at acute, convalescence and post-vaccination. (*B*) Frequency of N-specific and (C) spike-specific probe⁺ B cells at binned timepoints. (*D*) Isotype and (*E*) phenotypic analysis of N-specific probe⁺ B cells between convalescence and post-vaccination. (*F*) Isotype and (*G*) phenotypic analysis of spike-specific and N-specific probe⁺ B cells at binned timepoints. Statistical significance determined by (*A-C*) Dunn's multiple comparison test and (*D-G*) Tukey's multiple comparison's test. Any samples with <10 probe⁺ events are shown as open symbols. Only samples with 10 or more probe⁺ events are included in the phenotypic analysis.

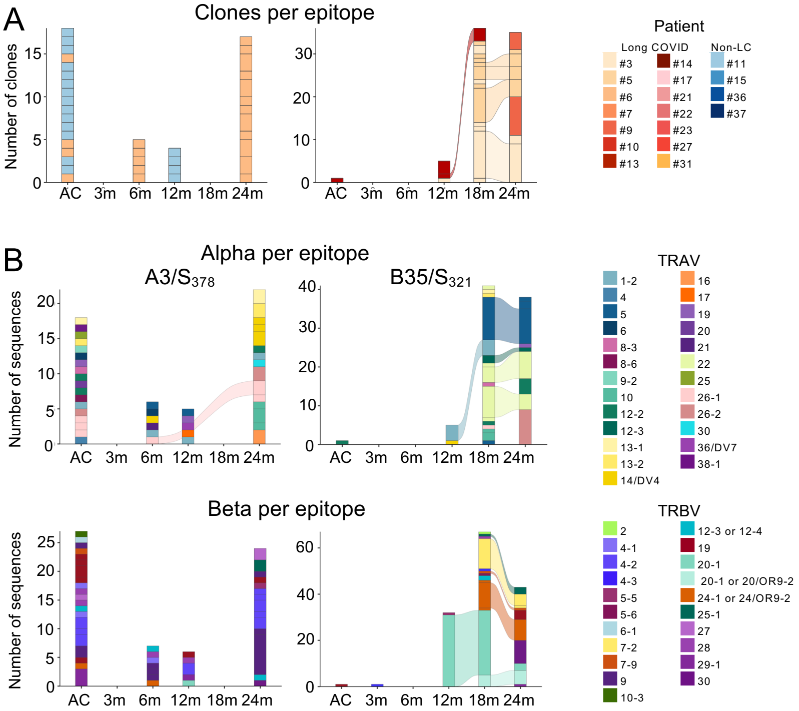


**Fig. S6.** A3/$S_{378}$- and B35/$S_{321}$-specific T cell receptor repertoires. (*A*) Combined long COVID and non-LC alluvial plots of A3/$S_{378}$ and B35/$S_{321}$ TCR repertoires, showing sharing of TCRαβ clones across the time-points by the band connections in colours representing individuals. (*B*) Alluvial plots of A3/$S_{378}$ and B35/$S_{321}$ TCR repertoires showing the sharing of TCRα and TCRβ CDR3 chains across the time points by the band connections in colours representing  and  usage.

**References**


1. COVID-19 Treatment Guidelines Panel (2023) Coronavirus Disease 2019 (COVID-19) Treatment Guidelines. (National Institutes of Health (NIH)).
2. T. H. O. Nguyen *et al.*, $CD8^+$ T cells specific for an immunodominant SARS-CoV-2 nucleocapsid epitope display high naive precursor frequency and TCR promiscuity. *Immunity* **54**, 1066-1082.e1065 (2021).
3. J. Kwok *et al.*, High resolution allele genotyping and haplotype frequencies for NGS based HLA 11 loci of 5266 Hong Kong Chinese bone marrow donors. *Hum Immunol* **81**, 577-579 (2020).
4. World Health Organization (2022) Post COVID-19 condition (long COVID). in *https://www.who.int/europe/news-room/fact-sheets/item/post-covid-19-condition#:~:text=Definition,months%20with%20no%20other%20explanation*.
5. J. R. Habel *et al.*, Suboptimal SARS-CoV-2−specific $CD8^+$ T cell response associated with the prominent HLA-A*02:01 phenotype. *Proc Natl Acad Sci USA* **117**, 24384-24391 (2020).
6. L. C. Rowntree *et al.*, SARS-CoV-2-specific T cell memory with common TCRαβ motifs is established in unvaccinated children who seroconvert after infection. *Immunity* **55**, 1299-1315.e1294 (2022).
7. T. H. O. Nguyen *et al.*, Robust SARS-CoV-2 T cell responses with common TCRαβ motifs toward COVID-19 vaccines in patients with hematological malignancy impacting B cells. *Cell Rep Med* **4**, 101017 (2023).
8. P. A. Mudd *et al.*, SARS-CoV-2 mRNA vaccination elicits a robust and persistent T follicular helper cell response in humans. *Cell* **185**, 603-613.e615 (2022).
9. W. Zhang *et al.*, Robust and prototypical immune responses toward COVID-19 vaccine in First Nations peoples are impacted by comorbidities. *Nat Immunol* **24**, 966-978 (2023).
10. C. E. van de Sandt *et al.*, Newborn and child-like molecular signatures in older adults stem from TCR shifts across human lifespan. *Nature Immunology* 10.1038/s41590-023-01633-8 (2023).
11. R Core Team (2022) R: A language and environment for statistical computing. in *R Foundation for Statistical Computing* (Vienna, Austria).
12. J. A. Juno *et al.*, Humoral and circulating follicular helper T cell responses in recovered patients with COVID-19. *Nature Medicine* **26**, 1428-1434 (2020).
13. C. Phetsouphanh *et al.*, Improvement of immune dysregulation in individuals with long COVID at 24-months following SARS-CoV-2 infection. *Nature Communications* **15**, 3315 (2024).
14. S. A. Valkenburg *et al.*, Molecular basis for universal HLA-A*02:01-restricted $CD8^+$ T-cell immunity against influenza viruses. *Proceedings of the National Academy of Sciences USA* 10.1073/pnas.1603106113, 201603106 (2016).
15. J. C. Brunson, Q. D. Read (2023) ggalluvial: Alluvial Plots in 'ggplot2'. in *R package version 0.12.5*.
16. Z. Gu, L. Gu, R. Eils, M. Schlesner, B. Brors, Circlize implements and enhances circular visualization in R. *Bioinformatics* **30**, 2811-2812 (2014).
17. P. Dash *et al.*, Quantifiable predictive features define epitope-specific T cell receptor repertoires. *Nature* **547**, 89-93 (2017).
18. K. Mayer-Blackwell *et al.*, TCR meta-clonotypes for biomarker discovery with tcrdist3 enabled identification of public, HLA-restricted clusters of SARS-CoV-2 TCRs. *Elife* **10** (2021).
